# Hydrogen exchange mass spectrometry reveals protein interfaces and distant dynamic coupling effects during the reversible self-association of an IgG1 monoclonal antibody

**DOI:** 10.1080/19420862.2015.1029217

**Published:** 2015-04-15

**Authors:** Jayant Arora, John M Hickey, Ranajoy Majumdar, Reza Esfandiary, Steven M Bishop, Hardeep S Samra, C Russell Middaugh, David D Weis, David B Volkin

**Affiliations:** 1 Department of Pharmaceutical Chemistry; Macromolecule and Vaccine Stabilization Center; University of Kansas; Lawrence, KS, USA; 2 Department of Formulation Sciences; MedImmune LLC; Gaithersburg, MD, USA; 3 Department of Chemistry and R.N. Adams Institute of Bioanalytical Chemistry; University of Kansas; Lawrence, KS, USA; # Present affiliation: Biopharmaceutical Research and Development; Lilly Research Laboratories; Eli Lilly and Company; Indianapolis, IN, USA

**Keywords:** reversible self-association, protein-protein interactions, monoclonal antibody, immunoglobulin G1, hydrogen exchange, mass spectrometry, flexibility, stability, aggregation, high protein concentration

## Abstract

There is a need for new analytical approaches to better characterize the nature of the concentration-dependent, reversible self-association (RSA) of monoclonal antibodies (mAbs) directly, and with high resolution, when these proteins are formulated as highly concentrated solutions. In the work reported here, hydrogen exchange mass spectrometry (HX-MS) was used to define the concentration-dependent RSA interface, and to characterize the effects of association on the backbone dynamics of an IgG1 mAb (mAb-C). Dynamic light scattering, chemical cross-linking, and solution viscosity measurements were used to determine conditions that caused the RSA of mAb-C. A novel HX-MS experimental approach was then applied to directly monitor differences in local flexibility of mAb-C due to RSA at different protein concentrations in deuterated buffers. First, a stable formulation containing lyoprotectants that permitted freeze-drying of mAb-C at both 5 and 60 mg/mL was identified. Upon reconstitution with RSA-promoting deuterated solutions, the low vs. high protein concentration samples displayed different levels of solution viscosity (i.e., approx. 1 to 75 mPa.s). The reconstituted mAb-C samples were then analyzed by HX-MS. Two specific sequences covering complementarity-determining regions CDR2H and CDR2L (in the variable heavy and light chains, respectively) showed significant protection against deuterium uptake (i.e., decreased hydrogen exchange). These results define the major protein-protein interfaces associated with the concentration-dependent RSA of mAb-C. Surprisingly, certain peptide segments in the V_H_ domain, the constant domain (C_H_2), and the hinge region (C_H_1-C_H_2 interface) concomitantly showed significant increases in local flexibility at high vs. low protein concentrations. These results indicate the presence of longer-range, distant dynamic coupling effects within mAb-C occurring upon RSA.

## Abbreviations

mAb: monoclonal antibody
BsAbs: bispecific antibodies
ADCs: antibody-drug conjugates
SC: subcutaneous
HX-MS: hydrogen exchange mass spectrometry
RSA: reversible self-association
IgG1: immunoglobulin G1
CDR: complementarity-determining regions
Fab: antigen binding fragment
Fc: crystallizable fragment
HC: heavy chain
LC: light chain
C_H_1-C_H_3: constant domains 1–3 respectively of the heavy chain
V_H_/V_L_: variable domain of the heavy/light chain
DLS: dynamic light scattering
CD: circular dichroism
SEC: size-exclusion chromatography
HPLC: high-performance liquid chromatography
BS^2^G: bis (sulfosuccinimidyl) 2,2,4,4 glutarate

## Introduction

Monoclonal antibodies (mAbs) and mAb derivatives are currently the fastest growing segment of the biopharmaceutical drug market.^1^ Antibody therapy often requires relatively high dosing (>1 mg/kg) that can be administered either by intravenous (IV) or subcutaneous (SC) injection. Due to higher costs, lower patient compliance, and longer administration times associated with IV drug delivery by medical professionals, the self-administration of mAb drugs by patients at home via SC administration is being extensively investigated.^2^ The formulation development challenges for SC administration of mAbs at high concentrations (∼100–150 mg/mL), for use in prefilled syringes and auto-injectors, include protein instability as well as injection volume limitations (less than 2 mL) due to high tissue backpressure or injection pain.^2^

Molecular interactions that occur in high concentration formulations can also introduce challenges to the development and manufacturing of mAbs. As the intermolecular distance between individual molecules decreases at higher protein concentrations, the extent of non-ideal behavior increases due to intermolecular interactions between the mAbs.^3,4^ Such intermolecular interactions increase the propensity of antibody molecules to undergo reversible self-association (RSA) where non-covalent multimers can form at high protein concentrations and then dissociate upon dilution.^5-7^ The RSA of mAbs presents various pharmaceutical challenges, including the formation of protein aggregation precursors that can lead to the irreversible formation of oligomers.^8,9^ In addition, the RSA of mAbs gives rise to a network of associated higher-order species that can affect the viscoelastic properties of the solution,^10^ resulting in increased viscosity,^5-7,11^ solution turbidity,^12^ and, under certain conditions, even phase transitions.^13^ The increase in solution viscosity also imposes manufacturing challenges including high back-pressure and clogging of membranes,^2^ as well as elevated levels of shear stress during pumping.^14^

Reversible-self association of mAbs is generally attributed to weak and transient non-covalent interactions (e.g., hydrogen bonding, electrostatic, hydrophobic, π-π, and van der Waals interactions) between antibody molecules. As the protein concentration increases and the intermolecular distances decrease, the extent of non-covalent interactions between molecules rises.^3,4^ Not only have certain regions in the antibody structure (i.e., fragment antigen-binding region (Fab) and fragment crystallizable region, Fc) been shown to interact at high protein concentrations,^15,16^ such transient interactions have been shown to involve specific amino acid residues and sequences within mAbs.^15,17^ In addition, varying solution conditions (e.g., ionic strength, pH, temperature, salt type, etc.) can either increase or decrease the extent of RSA of mAbs, depending on the distinct nature of the non-covalent interactions for an individual mAb formulated under specific conditions.^18,19^

A variety of analytical tools have been used to characterize the RSA of proteins, and related effects on solution properties, including dynamic light scattering (DLS),^21^ composition-gradient multi-angle static light scattering,^20,21^ isothermal titration calorimetry,^15^ surface plasmon resonance,^22^ proton magnetic relaxation dispersion,^23^ nuclear magnetic resonance,^24^ fluorescence resonance energy transfer,^25^ mass spectrometry (MS),^26^ self-interaction nanoparticle spectroscopy,^27^ size-exclusion chromatography,^28^ analytical ultracentrifugation,^29^ small angle X-ray scattering,^30^ and atomic force microscopy.^31^ Most of these measurements provide reliable data only at low-to-moderate protein concentrations (∼1–20 mg/mL). With further increases in protein concentration, the non-ideality of the solution, along with technical problems such as multiple scattering effects, compromises data reliability.^32,33^ NMR can provide higher resolution information about the site-specific nature of protein self-association, but the large size of antibody molecules and their complexes and the need for isotopic labeling limit the applicability of NMR to examine the RSA of antibodies. Such limitations have led to the widespread use of solution viscosity to indirectly monitor RSA of mAbs at high protein concentrations (∼20–200 mg/mL).^5,6,17^ Although viscosity measurements provide an experimentally convenient method to monitor RSA behavior of mAbs, they do not provide site-specific information about protein-protein interfaces.

Hydrogen exchange mass spectrometry (HX-MS) provides an exciting opportunity to obtain high-resolution information about higher-order structure in antibodies.^34-36^ For example, HX-MS has been recently applied to examine the effect of various solution factors and physicochemical structural changes on the local flexibility of mAbs, including salts from the Hofmeister series,^37^ pharmaceutical excipients,^38^ freeze-thaw cycles,^39^ methionine oxidation,^40^ asparagine deamidation,^41^ antibody-drug conjugation,^42^ deglycosylation and glycan modifications,^43^ and engineered point mutations.^44^

In this study, antibody clusters were characterized by a combination of DLS and chemical cross-linking experiments to determine the effect of solution conditions on the extent of RSA for an IgG1 mAb (referred to as “mAb-C”) as a function of mAb-C protein concentration (1–10 mg/mL). Solution viscosity measurements were then utilized to indirectly monitor the increase of RSA at higher mAb-C concentrations (up to 60 mg/mL protein). We then applied a novel HX-MS methodology, using a stable, lyophilized formulation followed by reconstitution in D_2_O solutions that promote RSA, to map RSA-induced changes in hydrogen exchange by mAb-C (i.e., comparing relatively low vs. high levels of RSA at 5 vs 60 mg/mL). This HX-MS analysis revealed specific peptide segments at the protein interface leading to RSA of mAb-C and also identified regions of mAb-C distant from the protein-protein interface with increased backbone flexibility.

## Results

### Defining solution conditions that favor RSA

**Figure 1.**
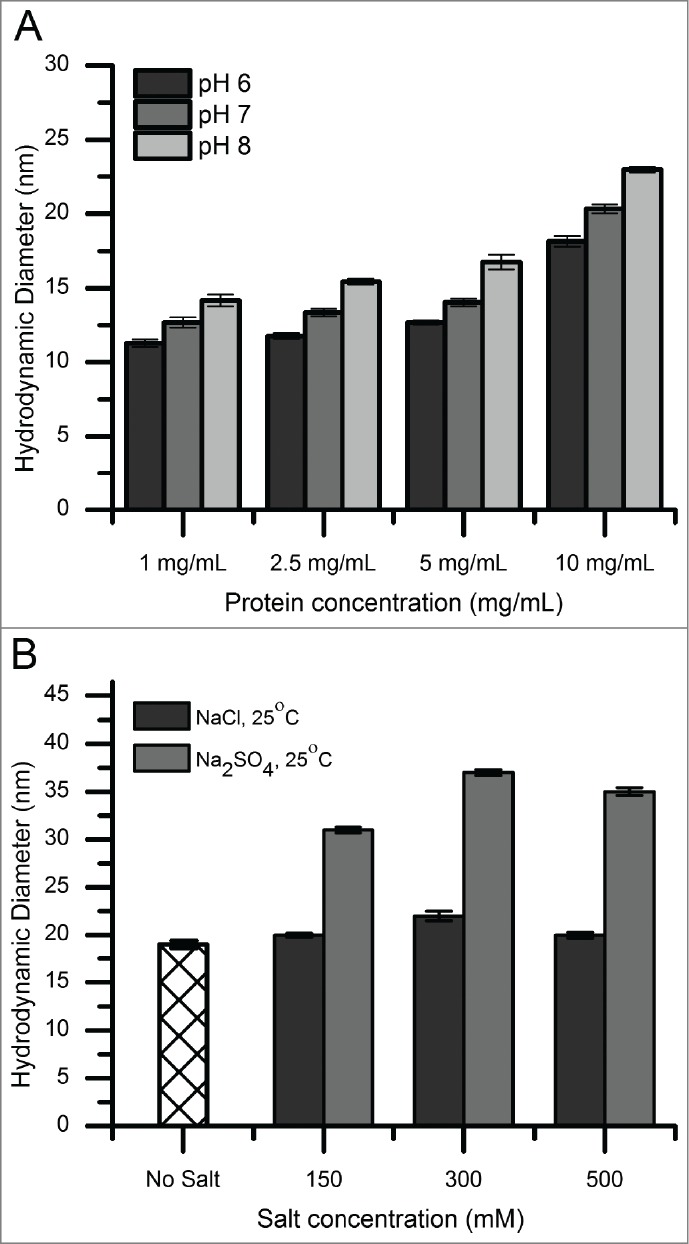
Hydrodynamic diameter of mAb-C under various solution conditions as measured by dynamic light scattering. (**A**) Effect of pH as a function of protein concentration. (**B**) Effect of salt type (NaCl and Na_2_SO_4_ and salt concentration. Experiments in panel **A** were conducted at 25°C with mAb-C samples prepared in 40 mM potassium phosphate buffer (pH 6, 7 and 8) containing 0.3 M NaCl. Experiments in panel **B** were conducted at 25°C with mAb-C samples prepared in 40 mM potassium phosphate buffer (pH 7) containing either NaCl or Na_2_SO_4_ at 0, 0.15, 0.3 and 0.5 M concentration. The error bars represent one standard deviation from 3 independent measurements.

DLS was used to measure the hydrodynamic diameter of mAb-C species under various solution conditions. **Figure 1** shows the effect of protein concentration (1–10 mg/mL), pH, salt concentration, and salt type on the size of mAb-C complexes. A solution of primarily monomeric mAb had a hydrodynamic diameter of ∼9–12 nm. The hydrodynamic diameter increased with increasing protein concentration, increased solution pH (**Fig. 1A**), and increased ionic strength (**Fig. 1B**). In addition, mAb-C formulated in a solution containing sodium sulfate showed increased hydrodynamic diameter compared to sodium chloride (**Fig. 1B**). These DLS results are consistent with those reported previously by Esfandiary et al. for the same antibody molecule formulated under similar solution conditions.^11,45^

**Figure 2.**
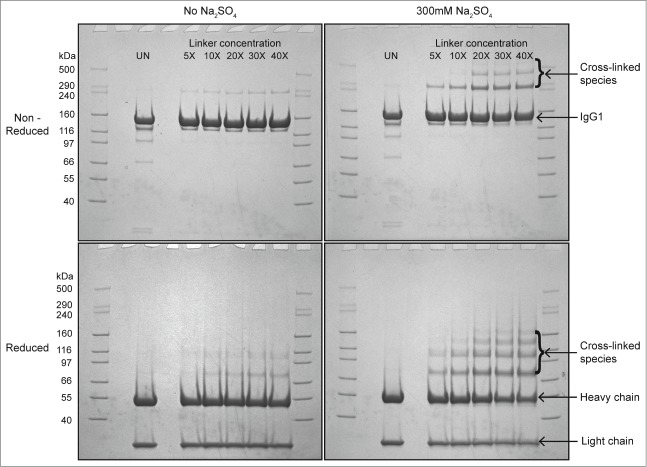
Cross-linking and SDS-PAGE analysis of reversible self-association (RSA) of mAb-C under different solution conditions. The mAb-C samples were prepared in 40 mM potassium phosphate buffer (pH 7) in the presence and absence of 0.3 M Na_2_SO_4_. The lane in each panel marked “UN” represents a mAb-C control with no added cross-linker. The first and last lane of each gel contains molecular weight standards. The masses are denoted on the left side gels. Subsequent lanes show the extent of mAb-C cross-linking in the presence of increasing molar ratio of BS^2^G cross-linker. Top-left panel, non-reduced SDS-PAGE gel showing cross-linking of mAb-C in the absence of Na_2_SO_4_. Top-right panel, non-reduced gel showing cross-linking of mAb-C in the presence of 300 mM Na_2_SO_4_. Bottom-left panel, reduced SDS-PAGE gel showing mAb-C in the absence of Na_2_SO_4_. Bottom-right panel, reduced gel of mAb-C in the presence of 300 mM Na_2_SO_4_. Cross-linking reactions were carried out at 4°C.

To further assess the effect of salt on reversible self-association (RSA) of mAb-C, we used chemical cross-linking to measure the effect of sulfate on the extent of RSA at pH 7. In the presence of 300 mM sodium sulfate, higher molecular weight bands at ∼300 kDa and ∼450 kDa were observed (**Fig. 2**, top panels). The intensity of these bands increased as the concentration of the cross-linker was increased from 5 to 40 molar excess over mAb-C. In the absence of sodium sulfate, only a very faint band appeared at ∼300 kDa and no band was observed at ∼450 kDa. Similar results were obtained from reduced samples of mAb-C (**Fig. 2**, bottom panels), where several higher molecular weight bands were observed above the heavy chain band (∼50 kDa).

The DLS and cross-linking results (**Figs. 1 and 2**) were limited to mAb-C concentrations of ∼1–10 mg/mL because higher concentrations led to experimental variability, non-ideal behavior or experimental artifacts (data not shown). The inability of such techniques to provide reliable sizing data at higher protein concentrations is consistent with previous reports.^2,46,47^ Despite analytical limitations of DLS at higher protein concentrations, the RSA-promoting conditions identified with DLS were selected for RSA studies at high protein concentrations. In order to characterize RSA at higher mAb-C concentrations (10–60 mg/mL), we turned to a combination of solution viscosity and HX-MS measurements, as described in the following sections.

### Solution viscosity as a function of protein concentration, temperature and salt concentration

**Figure 3.**
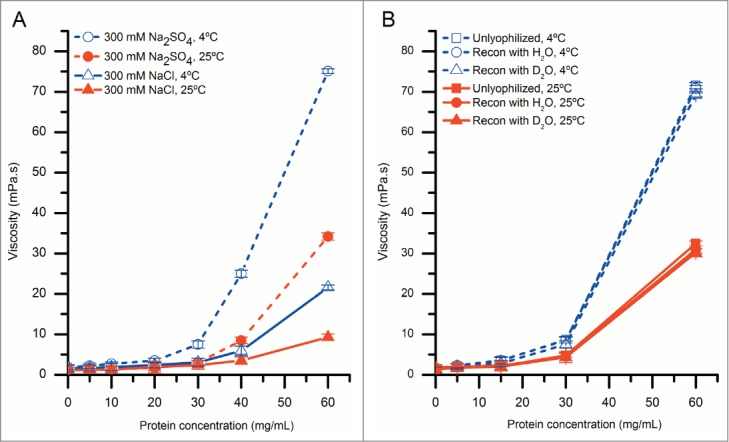
The effect of protein concentration, temperature and salt type on solution viscosity of mAb-C samples either before or after lyophilization and reconstitution. (**A**) Effect of temperature and salt type on viscosity of mAb-C as a function of protein concentration. (**B**) Effect of lyophilization and reconstitution diluent (with H_2_O- and D_2_O-based buffers) on the viscosity of mAb-C as a function of protein concentration. Samples of mAb-C for panel A were prepared in 40 mM potassium phosphate buffer (pH 7.0) containing 300 mM salt (NaCl or Na_2_SO_4_). For panel B, mAb-C samples that were not lyophilized were prepared in 40 mM potassium phosphate buffer (pH 7.0) containing 300 mM Na_2_SO_4_ and 10% (w/v) trehalose. Lyophilized samples were freeze-dried in 20 mM potassium phosphate buffer (pH 7.0) containing 10% (w/v) trehalose, and then reconstituted with either H_2_O or D_2_O buffers consisting of 20 mM potassium phosphate buffer (pH 7.0) and 300 mM salt Na_2_SO_4_. Viscosity measurements were taken either at 4°C or 25°C, as noted. The error bars represent one standard deviation from 3 independent measurements.

We used solution viscosity measurements to determine the effects of sulfate concentration, and temperature on RSA of mAb-C at higher protein concentrations. The solution viscosity of mAb-C samples increased with protein concentration and was further elevated in the presence of sodium sulfate and at lower temperature (**Fig. 3A**). For example, the solution viscosity of mAb-C at protein concentrations ranging from 5 mg/mL to 60 mg/mL at 4°C varied from 1 mPa·s to ∼75 mPa·s in the presence of 300 mM sodium sulfate, and from 1 mPa·s to ∼20 mPa·s in the presence of 300 mM sodium chloride. The same trends were also present at 25°C, but at lower viscosity values (**Fig. 3A**).

Based on the combined results from the DLS, chemical-crosslinking and solution viscosity experiments, the subsequent evaluation of the effects of RSA on the local flexibility of mAb-C (as measured by HX-MS analysis) was performed under the following conditions: 2 protein concentrations (i.e., 5 and 60 mg/mL) were prepared in a deuterated phosphate buffer at pH 7 containing 300 mM sodium sulfate and 10% trehalose. Elevated solution pH and salt levels (and salt type) amplified the propensity of mAb-C to reversibly self-associate (as shown above) while the sugar was needed as a lyoprotectant (see below). The 5 mg/mL mAb-C was selected as a control with relatively limited RSA, whereas the 60 mg/mL mAb-C sample was chosen as a sample displaying more extensive RSA. Esfandiary et al. have recently shown that the same mAb can form a monomer-trimer-hexamer equilibrium under similar solution conditions (at 1–10 mg/mL at room temperature).^45^ Nonetheless, as shown in this work, notable differences in the extent of RSA are observed by solution viscosity and HX-MS measurements at 5 vs. 60 mg/mL of mAb-C.

### Development of a freeze-dried formulation for HX-MS analysis of RSA

A conventional hydrogen exchange experiment typically begins with a five- to twenty-fold dilution of the protein with D_2_O, but in our case, such a dilution would alter the reversible self-association of mAb-C. To maintain high protein concentration during hydrogen exchange, we developed an approach based on reconstitution of lyophilized protein with D_2_O. To evaluate the effects of lyophilization and reconstitution on mAb-C we used viscosity measurements, CD, and SEC. mAb-C was prepared at concentrations between 5 and 60 mg/mL in phosphate buffer (pH 7.0) containing 10% trehalose (w/v). Samples were then freeze-dried and reconstituted and compared with samples that had not been freeze-dried.

A comparison of the solution viscosity of the reconstituted mAb-C samples with control mAb-C samples that were not lyophilized is shown in **Figure 3B**. There was no difference between the viscosity of the control (no freeze-drying) and lyophilized/reconstituted mAb-C samples, and no difference in the viscosity between samples reconstituted with either H_2_O or D_2_O at both temperatures (4°C and 25°C). These results show that the lyophilization/reconstitution of mAb-C at different protein concentrations, in either H_2_O or D_2_O buffers, had no significant effect on the extent of RSA of mAb-C, as measured by solution viscosity. This experimental approach thus affords the opportunity to prepare low and high protein concentration solutions of mAb-C in D_2_O-containing buffers for HX-MS analysis.

**Figure 4.**
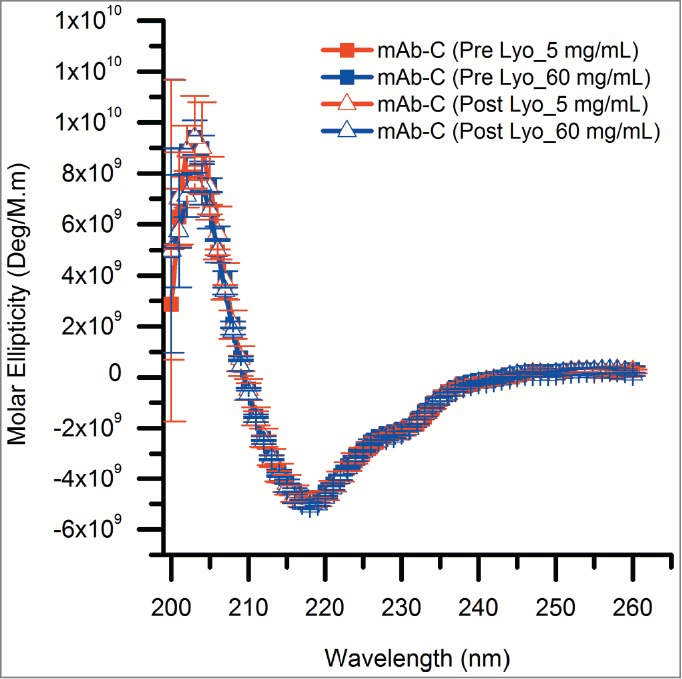
Circular dichroism spectra showing the effect of the lyophilization process on the overall secondary structure of mAb-C. All mAb-C samples (pre and post lyophilization) were diluted to 0.3 mg/mL with 40 mM potassium phosphate buffer (pH 7.0) containing 300 mM Na_2_SO_4_ and 10% (w/v) trehalose for analysis at 10°C. The error bars represent one standard deviation from 3 independent measurements.

To further ensure that freeze-drying had no notable effects on the overall structural integrity of mAb-C, lyophilized mAb-C samples were reconstituted with D_2_O-based reconstitution buffer and analyzed by both circular dichroism (CD) and size-exclusion chromatography (SEC). Far-UV CD spectra of control and lyophilized mAb-C samples are indistinguishable with minima at 217 nm (**Fig. 4**), characteristic of the high beta sheet content of IgG domains. This result indicates that lyophilization followed by reconstitution did not induce any changes in the overall secondary structure content of mAb-C.

The aggregate content of the same samples was measured by SEC (**Table 1**). The amount of soluble aggregates and fragments present in the mAb-C samples did not change after lyophilization, but the amount of insoluble aggregates (loss of total area by SEC) after reconstitution was somewhat higher (2.1%–2.7%) compared to control samples. Based on these results, the small amount of aggregate was removed by centrifugation prior to HX-MS experiments (see Methods). In addition, the overall structural integrity of mAb-C before and after lyophilization and reconstitution was further confirmed by HX-MS analysis as described below. These results indicate that lyophilization followed by reconstitution did not induce significant aggregation.

**Table 1.** Effect of lyophilization and reconstitution on the aggregation profile of mAb-C as measured by size-exclusion chromatography (SEC). Samples of mAb-C were prepared at 5 and 60 mg/mL in 20 mM potassium phosphate buffer (pH 7.0) with 10% (w/v) trehalose (pre-lyophilization samples). After lyophilization, mAb-C samples were reconstituted with D_2_O-based 20 mM potassium phosphate buffer (pH 7.0) with 300 mM Na_2_SO_4_ (post-lyophilization samples). The experimental data are mean and standard deviation calculated from three independent measurements on three separate lyophilized vials.

mAb-C Sample	% Insoluble aggregates	% Monomer	% Soluble aggregates	% Fragments
5 mg/mL, pre-lyophilization	0.0 ± 0.1	99.4 ± 0.1	0.5 ± 0.1	0.1 ± 0.1
5 mg/mL, post-lyophilization	2.1 ± 0.9	97.3 ± 0.9	0.5 ± 0.1	0.1 ± 0.1
60 mg/mL, pre-lyophilization	0.0 ± 0.1	99.4 ± 0.1	0.5 ± 0.1	0.1 ± 0.1
60 mg/mL, post-lyophilization	2.7 ± 0.2	96.7 ± 0.3	0.5 ± 0.1	0.1 ± 0.1

### Effects of reversible self-association (RSA) on hydrogen exchange of mAb-C

**Figure 5.**
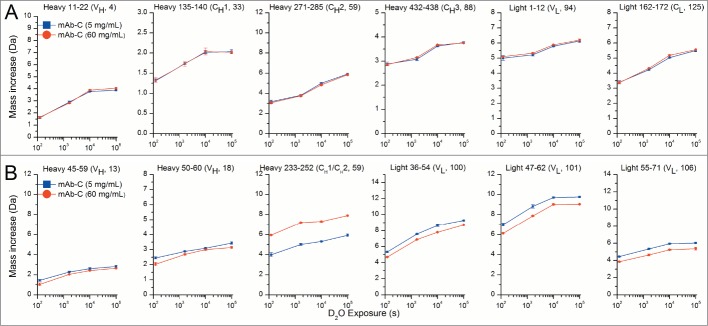
Deuterium uptake by 12 representative peptide segments from mAb-C measured at 5 and 60 mg/mL as determined by HX-MS. (**A**) Six representative peptides that showed no differences in hydrogen exchange kinetics between low and high protein concentrations. (**B**) Six representative peptides that showed significant changes in hydrogen exchange kinetics between low and high protein concentrations. Domain location and peptide number of the segment are shown in parentheses. The error bars represent one standard deviation from 3 independent experiments. Refer to **Figure S2** for deuterium uptake plots of all 130 peptides in the data set.

The RSA of mAb-C was analyzed by HX-MS by reconstituting 5 and 60 mg/mL lyophilized mAb-C preparations with a D_2_O-based reconstitution/labeling buffer. Samples were incubated in D_2_O for varying periods of time, quenched, digested with pepsin, and the deuterium uptake in pepsin-generated peptides of mAb-C was measured by MS (see Methods). A total of 130 mAb-C peptides were reproducibly generated by pepsin digestion, resulting in sequence coverage of 94% for the heavy chain and 93% for the light chain of mAb-C (see **Fig. S1**). **Figure 5** shows representative deuterium uptake results for several different peptides as a function of hydrogen exchange labeling time. **Figure 5A** presents results from some representative mAb-C peptides that show no significant differences in hydrogen exchange kinetics between RSA and non-RSA mAb-C. In contrast, **Figure 5B** contains examples where RSA induced significant differences in hydrogen exchange kinetics: RSA caused both faster and slower hydrogen exchange in different regions of mAb-C. As discussed in more detail below, for approximately 90% of the peptides, however, there were no significant differences in deuterium uptake between RSA and non-RSA mAb-C based on our significance criteria (see Methods).

**Figure 6.**
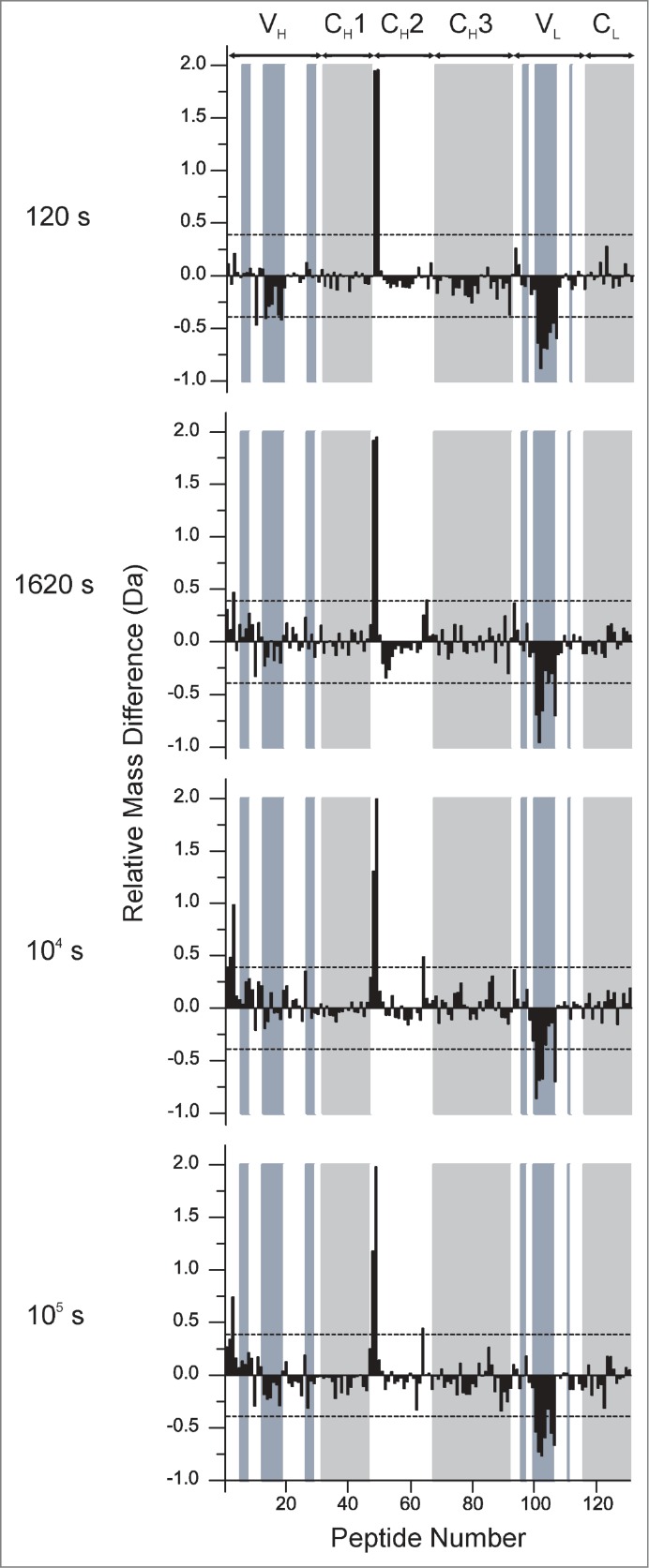
Relative differences in deuterium uptake at 4 exposure times as measured by HX-MS for 130 peptide segments of mAb-C at 60 mg/mL vs. 5 mg/mL at pH 7.0. The individual peptides are arranged on the horizontal axis starting from the N-terminal of the heavy chain and ending at the C-terminal of the light chain. The peptides are numbered sequentially based on the locations of their middle residues (see Table S1 for the identities and locations of the peptides). The horizontal axes of these plots denote the peptide numbers from 1 to 130. The vertical axis is the difference between exchange at 60 mg/mL vs. 5 mg/ml: $\Delta m\left(t\right)=m_{60}\left(t\right)–m_{5}\left(t\right)$. Positive bars indicate an increase in deuterium uptake for a particular peptide segment at high protein concentration (60 mg/mL) and negative bars indicate decreased deuterium uptake for a peptide segment at 60 mg/mL compared to lower protein concentration (5 mg/mL). The dashed lines at ± 0.4 Da indicate the 99% confidence limits for significant differences. White and gray shades in the background of the figure represent IgG domain boundaries and each domain is labeled at the top of the figure. Shades in blue represent CDR segments on mAb-C. Segment locations in the mAb-C sequence and their corresponding peptide numbers can be found in Table S1 in the supporting information. An average of 3 independent mass measurements was used to calculate each mass difference data point corresponding to all the exposure time-points.

**Figure 6** presents a global view of these data by presenting the hydrogen exchange differences across the 130 peptides generated in a single plot. The differences in hydrogen exchange between mAb-C samples at 60 mg/mL and 5 mg/mL: $\left[\Delta m\left(t\right)=m_{60}\left(t\right)-m_{5}\left(t\right)\right]$ are plotted on the vertical axis. The individual peptides are arranged on the horizontal axis starting from the N-terminal of the heavy chain and ending at the C-terminal of the light chain. The peptides are numbered sequentially based on the locations of their middle residues (see **Table S1** for the identities and locations of the peptides). The domain locations are indicated by labels and alternate shading in white and gray. These plots efficiently display the trends in local flexibility changes between the RSA and non-RSA mAb-C. The direction of the bar indicates whether an individual peptide becomes more flexible (Δm(t)>0 Da) or less flexible (Δm(t)<0 Da) upon RSA. The dashed lines in **Figure 6** indicate Δm(t) values that exceed the 99% confidence limit of ±0.4 Da for statistically significant changes induced by RSA (see Methods).

The results in **Figure 6** reveal differences in hydrogen exchange in mAb-C at high vs. low concentrations of mAb-C, conditions shown to affect the RSA of the antibody. Most of the mAb-C peptides (>90%) show no significant differences in hydrogen exchange. The specific peptides of mAb-C that display significant decreases in hydrogen exchange (Δm(t)<0.4 Da) at high vs. low concentrations of mAb-C are located in V_H_ and V_L_ domains, primarily in sequences that include the CDR2H and CDR2L. For example, in the V_H_ domain, there is a 26 amino acid sequence (HWVRQAPGQGLEWMGWINPHSGGTNY) that spans the CDR2H sequence of mAb-C and is covered by 3 V_H_ domain peptides (peptide numbers 10, 13 and 18 corresponding to HC 35–59, HC 45–59 and HC 50–60, respectively). These three peptides display significant decreases in hydrogen exchange at the 120 s time point. The magnitude of these effects is small, |Δm|<0.5 Da. In the V_L_ domain, there is a 36 amino acid sequence covering LC 36–71 (YQQKPGKAPKLLIYVASSLQSGVPSRFSGSGSGTDF), corresponding to peptide numbers 100, 101, 102, 103, 104, 105 and 106, where significant decreases in hydrogen exchange in high vs. low concentrations of mAb-C were also observed. These seven peptides showed differences across all of the deuterium exposure time points. In the light chain, the magnitudes of the effects, |Δm|>0.7 Da, are much larger than the effects in the heavy chain. The LC 36–71 sequence in the V_L_ domain spans the CDR2L sequence of mAb-C. In summary, significant decreases in hydrogen exchange (i.e., increased protection against deuterium uptake) were observed upon RSA of mAb-C in 2 of the 6 CDR regions in the mAb (i.e., the CDR2 region of the heavy and light chain).

Interestingly, several other peptide segments from mAb-C concomitantly displayed the opposite effect: increased local flexibility (Δm(t)>0.4 Da) at the higher (vs. lower) mAb-C concentration (**Fig. 6**). For example, 2 peptides in the V_H_ domain covering HC 4–29 (GAEVKKPGASVKVSCKASGYTF, corresponding to peptide number 3 and 4) showed this trend. In addition, in comparing the 60 mg/mL vs. 5 mg/mL samples of mAb-C, 2 peptide segments located in the interface of C_H_1 and C_H_2 domains covering HC 229–252 (DKTHTCPPCPAPELLGGPSVFLFPPK, corresponding to peptide number 48 and 49) as well as one C_H_2 domain peptide covering HC 311–325 (VSVLTVLHQDWLNGK, corresponding to peptide 64) also showed significant increases in deuterium uptake at one or more time points.

**Figure 7.**
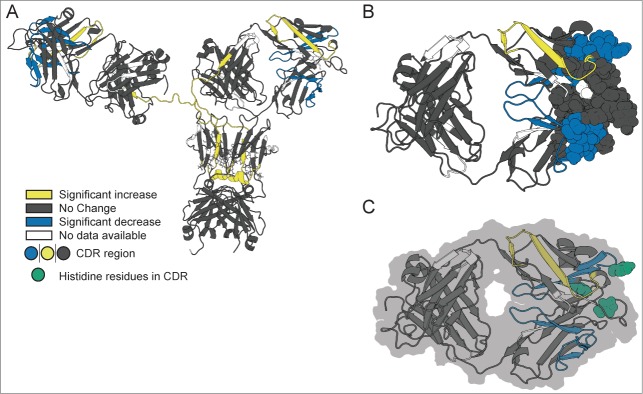
Effect of concentration-dependent RSA on deuterium uptake of various segments of mAb-C as measured by HX-MS plotted onto a homology model of mAb-C. **(A)** Entire mAb, **(B)** view of the Fab domain and **(C)** view of the Fab domain with histidine residues in the peptide segments containing the CDR2H and CDR3H sequences highlighted in green. Changes in deuterium uptake of particular peptide segments are colored according to the legend and are derived from the differential exchange data shown in **Figure 6**.

**Figure 7** further summarizes these HX-MS results as mapped onto a homology model of mAb-C (**Fig. 7A** shows the entire mAb-C molecule and **Figure 7B** and **C** displays a close-up view of the CDR2 regions within the Fab domain). The peptide segments in mAb-C where RSA caused significant decreases in hydrogen exchange (Δm(t)<−0.4 Da) are colored blue, while regions that exhibited significant increases (Δm(t)>0.4 Da) are colored yellow. The regions of mAb-C without significant effects (|Δm(t)|≤0.4 Da) are colored in gray and regions of mAb-C lacking hydrogen exchange data are shown in white. The peptides that exhibited decreased hydrogen exchange at high vs. low mAb-C concentration constitute the primary protein-protein interface for RSA of mAb-C. The peptide segments showing increased local flexibility may indicate long-range dynamic coupling effects of RSA in mAb-C (see Discussion).

Finally, to further confirm that the lyophilization/reconstitution method did not adversely affect the structural integrity of mAb-C, we measured hydrogen exchange kinetics before and after lyophilization at 2 protein concentrations (6 and 60 mg/mL) in a subset of 35 peptides covering all domains of mAb-C and including all peptides that had significant differences in local flexibility caused by RSA (**Fig. 6**). There were no significant differences in hydrogen exchange kinetics between lyophilized and control mAb-C samples at either protein concentration (see **Figs. S4 and S5**).

## Discussion

The main aim of this study was to develop an HX-MS method to characterize the protein interfaces involved with the reversible self-association (RSA) of mAb-C directly at high protein concentrations (i.e., 60 mg/mL). Commonly available biophysical measurements used to monitor RSA not only lack such local sequence information, but can only be performed at lower protein concentrations (∼10 mg/ml) due to solution non-ideality (see Introduction). These analytical limitations were encountered with DLS and SV-AUC measurements of mAb-C under different solution conditions as described in this work and as reported previously.^11,45^ In the present study, we correlate solution effects on the RSA of mAb-C, as determined by DLS and chemical crosslinking at lower mAb-C concentrations, to solution viscosity measurements at higher protein concentrations. We then directly mapped the local regions of mAb-C involved at the interface of RSA at the higher protein concentration using HX-MS, an analytical tool that has been widely used to map interfaces of protein-protein interactions.^48-50^

### Effect of solution conditions on RSA of mAb-C

As an initial evaluation of the RSA of mAb-C, we employed DLS and chemical cross-linking. There was a measurable increase in the RSA of mAb-C with increasing protein concentration, solution pH, and ionic strength (**Figs. 1 and 2**). These results are consistent with trends previously reported for the RSA of mAb-C at low protein concentrations by DLS, SV-AUC and CG-MALS.^11^ Many of these same trends were apparent at higher protein concentrations, as revealed by changes in viscosity, where an exponential increase in viscosity was observed ranging from ∼1 to ∼75 mPa·s depending on the protein concentration and solution conditions (**Fig. 3**). Increases in intermolecular associations between antibody molecules at high protein concentrations have been previously correlated to elevated solution viscosity values.^5,51^ This effect can be attributed to the antibody network formation at high protein concentration, which affects the packing volume fraction of the antibody and ultimately results in an increase in solution viscosity.^10^ For example, Pathak et al. demonstrated that the presence of reversibly associated clusters at high protein concentrations contributed to an increase in solution viscosity.^52^

Similar trends in viscosity in response to changes in solution conditions that we describe here for mAb-C have been reported for other IgG1 mAbs, where the extent of viscosity increased in a concentration-dependent manner with increasing ionic strength ^11,18,30,53,54^ and solution pH,^11,55^ related to elevated levels of protein RSA due to charge shielding effects.^56-58^ The isoelectric point (pI) range of mAb-C is basic (pI∼9.1–9.4),^45^ and therefore, the overall surface charge of mAb-C is expected to be positive at neutral pH. As the pH was changed from 6 to 8, the tendency of mAb-C to self-associate increased, possibly due to overall decrease in electrostatic repulsive interactions. In addition, more specific charge effects are possible, including protonation/deportation of histidine residues upon a change in solution pH in the range of the pKa (∼ pH 6). From our present work, there are 2 histidine residues in the 26 amino acid sequence covering the CDR2H region (HWVRQAPGQGLEWMGWINPHSGGTNY) that showed significantly decreased hydrogen exchange upon RSA of mAb-C. The probable involvement of histidine side chain residues in the RSA of this mAb was demonstrated indirectly by DLS studies (vs. solution pH and composition) as reported recently.^11,45^ The homology model indicates that one of these histidines is highly solvent exposed (see **Fig. 7C**).

As electrostatic repulsive interactions decrease when solution pH approaches pI, other non-covalent attractive interactions such as hydrophobic and van der Waals interactions are expected to become more dominant allowing mAb-C monomers to self-associate to a greater extent. To further explore the effects of 2 anions, sulfate and chloride, on the extent of RSA of mAb-C, both hydrodynamic diameter and solution viscosity of mAb-C samples were measured in the presence of both the anions. Sulfate had a bigger effect on the extent of RSA of mAb-C than chloride (**Figs. 1B** and **3A**). In terms of ranking in the Hofmeister series of anions, divalent sulfate anions have a stronger kosmotropic effect on proteins than monovalent chloride ions.^59^ Sulfate ions interact more strongly with the positively charged amino acid side chains on a protein surface than chloride ions,^60,61^ presumably resulting in enhanced charge shielding effects. Sulfate ions can also desolvate polar and non-polar regions of the protein surface, thus aggravating hydrophobic interactions by decreasing protein solubility, an effect commonly known as “salting out.”^62,63^ These ion-protein interactions correlate well with our observations of enhanced RSA of mAb-C in the presence of sulfate anions. Esfandiary et al. demonstrated by modeling of light scattering data that mAb-C formulated in the presence of 150 mM sodium sulfate at room temperature can assemble into monomer-trimer-hexamer mixtures, ranging from 100% monomer to ∼75%-20%-5% molar ratios, as the protein concentration increases from 1 to 10 mg/mL. ^11,45^

### Development of an HX-MS method to examine RSA

In most HX-MS studies on protein-protein interactions, the interactions are typically non-reversible or have relatively high affinity; therefore, HX-MS experiments can easily be carried out at low protein concentrations after dilution of protein stock solutions with deuterium-containing buffers. In contrast, RSA is a concentration-driven phenomenon. In order to study RSA of mAbs, HX-MS experiments need to be performed at high protein concentrations requiring a novel methodology to prepare high protein concentrations in deuterium-containing buffers. To this end, antibody solutions under RSA-promoting solution conditions at high and low protein concentration were lyophilized and reconstituted with a D_2_O-based labeling/reconstitution buffer.

Lyophilization of proteins can cause detrimental effects on physical stability through ice crystal-water interfaces, cold-denaturation, solution pH change during lyophilization, and dehydration stress.^64,65^ We used a combination of CD and SEC analysis to demonstrate that the lyophilization process itself did not lead to structural alterations or aggregation of mAb-C (**Fig. 4** and **Table 1**). In addition, based on hydrogen exchange measurements of 35 key peptides from mAb-C (**Figs. S4 and S5**), we can conclude that lyophilization followed by reconstitution did not cause any significant changes in the local flexibility across the mAb-C molecule (**Fig. S3**). These results illustrate a potential new pharmaceutical application of HX-MS: evaluations of the structural integrity of protein samples before and after lyophilization and reconstitution. These results also demonstrate the reproducibility achievable with this lyophilization/reconstitution HX-MS approach.

### HX-MS mapping of the protein interface of RSA of mAb-C

From the HX-MS analysis of mAb-C at low and high protein concentrations (**Figs. 5** and **6**), peptides with increased protection against deuterium uptake (i.e., slowed hydrogen exchange) at high mAb-C concentration are assigned as the primary interface for RSA of mAb-C. Two regions in V_L_ and V_H_ (LC 36–71 and HC 35–60) showed a significant decrease in hydrogen exchange at high mAb-C concentration. Both of these regions cover the CDR2 region in the heavy and light chain of mAb-C, respectively, which demonstrates 2 of the 6 CDRs in mAb-C are involved with RSA. The involvement of CDR regions, including specific aromatic/hydrophobic residues, in RSA of antibodies has been reported in previous studies with other antibodies.^16,66,67^

More specifically with mAb-C, LC 36–71 and HC 35–60, which span CDR2L and CDR2H, respectively, showed significant decreases in hydrogen exchange under RSA-promoting conditions (**Fig. 7**). This result indicates that the amino acid residues encompassing the CDR2 sequence within the Fab region of mAb-C provide an interface for RSA at high protein concentrations. More specifically, protection in the light chain was detected in 7 overlapping peptides spanning CDR2L in the V_L_ domain of mAb-C, LC 36–71 (YQQKPGKAPKLLIYVASSLQSGVPSRFSGSGSGTDF). In the heavy chain, protection was detected in 2 peptides spanning CDR2H in the V_H_ domain, for HC 35–60 (HWVRQAPGQGLEWMGWINPHSGGTNY). CDR loops are naturally hypervariable, provide a unique identity to each mAb, and are solvent exposed for high affinity binding to the antigen. The sequences that became significantly protected at 60 mg/mL (i.e., upon more extensive RSA of mAb-C) contain numerous aromatic/hydrophobic residues. The LC 36–71 sequence contains 4 aromatic residues (Y and F) and 6 aliphatic residues (I, L, V) and the HC 35–60 sequence has 4 aromatic (Y and W) and 3 aliphatic residues (I, L, V). In addition, some of the peptide segments covering these sequences also contain histidine as well as charged amino acid residues at pH 7. Since the addition of 300 mM sodium sulfate promotes the RSA of mAb-C, the charged residues may become more shielded or charge-neutralized by sulfate binding. Upon such reduced electrostatic repulsive interactions, the presence of aromatic and hydrophobic residues can potentially facilitate RSA of mAb-C at high protein concentrations. Protection from hydrogen exchange in the V_L_ segment is stronger than in the V_H_ segment suggesting that there is higher affinity in the V_L_ segment.

To the best of our knowledge, this is the first study that utilizes HX-MS to characterize RSA of antibodies at peptide-level resolution directly at high protein concentrations. Several research groups have made observations using lower resolution biophysical tools to identify specific regions in an antibody associated with RSA. For example, Kanai et al. measured solution viscosity of purified F(ab’)_2_ and Fab fragments of a self-associating IgG1 mAb and concluded that the interface of RSA was in the Fab region.^6^ Yadav et al. swapped charged residues in the CDR region of a self-associating IgG1 mAb with those of a non-self-associating antibody and observed a significant decrease in solution viscosity and weight average molecular weight (M_wc_).^7^ Using these same mutants, the authors of a separate study used coarse-grained modeling to link domain-level charge distribution in the Fab region of the antibody to RSA.^68^ In another study, substitution of aromatic residues with non-aromatic amino acids (F99A, W100A) in the CDR3H region of the antibody caused a considerable decrease in RSA and an increase in protein solubility.^16,67^ However, Fab-Fab interactions are not necessarily always responsible for such interactions between antibody molecules. For example, Nishi et al. reported Fc-mediated RSA of an antibody under low ionic strength solution conditions.^15^

Several groups have examined the protein-protein interfaces of irreversible antibody aggregates (i.e., dimers and oligomers) by HX-MS. For example, Zhang et al. used HX-MS analysis of purified mAb aggregates to show that Fab-Fab interactions in the CDR region were generated as part of irreversible aggregate formation caused by heat exposure.^39^ In addition to HX-MS, antibody aggregates have been characterized using other higher resolution techniques. For example, Deperalta et al. used hydroxyl radical footprinting to map the interface region of an antibody dimer. This work demonstrated that the protein-protein interface lies in the Fab domain of the antibody.^69^ Using alternative approaches, Paul et al. used transmission electron microscopy to visualize purified antibody dimers and suggested Fab-Fab interactions were responsible for association.^70^ Wang et al. used computational predictive tools to delineate aggregation-prone regions in variable domains of an antibody located in or around the CDR region.^71^ A recent study by Iacob et al. showed that irreversible antibody aggregation can affect the flexibility of the mAb's hinge loop region. They demonstrated decreased local backbone flexibility in the hinge region upon formation of a disulfide cross-linked mAb dimer.^72^ Interestingly, as discussed below, the opposite effect (i.e., increased backbone flexibility in several regions including the hinge loop) was observed in our present work upon RSA of mAb-C.

### HX-MS mapping of distant dynamic effects on other regions of mAb-C upon RSA

We also observed a significant increase in hydrogen exchange in regions in mAb-C that are distant from the RSA protein-protein interaction site described above (see **Figs. 6** and **7**). These changes were observed in regions covering HC 4–29 (V_H_ domain, peptide number 2 and 3), HC 229–252 (C_H_1-C_H_2 interface, peptide number 48 and 49) and HC 311–325 (C_H_2 domain, peptide number 64). Among these changes, an increase in local flexibility at the hinge region (C_H_1-C_H_2 interface) of the antibody had the largest magnitude. One way to explain this significant increase in local backbone flexibility in the hinge region is through long-range, distant dynamic coupling effects that may occur upon protein-protein interactions.

In biological systems, protein allosteric effects play a major role in cellular regulation. The classical model of allosteric conformational effects constitute binding of a ligand to a region or domain of a protein and its effect on conformation of a distal, functional region of the protein.^73,74^ An emerging view of protein allostery includes a dynamic continuum in which protein-protein or protein-ligand interactions result in propagation of a signal through changes in protein dynamics, either with or without large scale conformational changes.^73,74^ Such alterations in protein dynamics as part of allosteric regulation of proteins are sometimes referred to as propagation of an “allosteric wave.” ^75^ Examples of such flexibility shifts within single protein molecule upon ligand binding or protein-protein interaction have been reported.^76,77^ Such changes in protein dynamic allostery can also occur upon post-translational modifications, or upon changes in solution pH or protein concentration.^75^

Although the distant dynamic coupling effects we observed within mAb-C upon extensive RSA may lack any biological consequences, the molecular mechanism of alterations in local backbone flexibility upon protein-protein interactions are “allosteric-like” in that they resemble the molecular mechanisms of protein dynamic allostery.^73,74,78^ The observed localized changes in backbone dynamics upon RSA may potentially have important implications in terms of pharmaceutical properties of a mAb including storage stability, manufacturability, and syringeability. Since HX-MS provides increased resolution for characterizing RSA directly at high protein concentrations, this information could be used to design superior next-generation mAb molecules with lower propensity for RSA. In terms of future work, further establishing the universality of such behavior among self-associating mAbs is being evaluated in our laboratories with a series of mAbs that reversibly self-associate to varying extents.

## Materials and Methods

### Materials

A highly purified IgG1 mAb at 10 mg/mL, referred to as “mAb-C,” was produced by MedImmune LLC, Gaithersburg, MD. The antibody stock solution was dialyzed into various buffers as indicated below. LC-MS grade water, 2-propanol and dibasic anhydrous potassium phosphate (>99.0%) and sodium chloride were obtained from Fisher Scientific (Fair Lawn, NJ). LC-MS grade acetonitrile was purchased from Honeywell (Morristown, NJ). Formic acid (≥99.0% LC-MS-grade) was purchased from Thermo Scientific (Rockford, IL). Monobasic anhydrous potassium phosphate (99.5%), sodium sulfate (>99.0%), porcine pepsin, tris (2-carboxyethyl) phosphine hydrochloride (TCEP), guanidine hydrochloride, deuterium oxide (99.9% D) and tris (hydroxymethyl) aminomethane hydrochloride (≥99.0%) were purchased from Sigma-Aldrich (St. Louis, MO). The reagent bis-(sulfosuccinimidyl) 2,2,4,4, glutarate-d_0_(BS^2^G-d_0_) was obtained from Pierce Biotechnology (Rockford, IL). The disaccharide α,α-trehalose dihydrate was obtained from Ferro Pfanstiehl laboratories (Waukegan, IL).

## Methods

### Sample preparation

As needed, mAb-C samples were dialyzed using 3.5 kDa molecular-weight-cutoff membranes (Slide-A-Lyzer, Thermo Scientific, Rockford, IL). mAb-C samples were concentrated using Vivaspin-20 (10 kDa molecular weight cutoff) ultrafiltration columns (Sartorius Stedim, Gottingen, Germany).

### Dynamic light scattering

Stock samples of mAb-C were dialyzed against 40 mM potassium phosphate buffer (pH 6–8) containing either NaCl or Na_2_SO_4_ at concentrations ranging from 0.15 to 0.5M overnight at 4°C. Dialyzed samples were then diluted to 1, 2.5, 5 and 10 mg/mL using the dialysis buffer. The concentration of mAb-C was determined using ultraviolet absorption spectroscopy (A_280 nm_) using E_1 cm_ 0.1% of 1.54 mL mg^−1^ cm^−1^.^45^ DLS experiments were performed in triplicate at 25°C using the DynaPro Plate Reader (Wyatt Technology, Santa Barbara, CA). Scattered light was analyzed using a backscatter detector fixed at an angle of 173°. An autocorrelation function was calculated using a built-in digital autocorrelator. 20 μL of sample was added to each well of a 384-well plate, and 10 μL of paraffin oil was added on top of each sample to inhibit sample loss by evaporation. The 384-well plate was centrifuged at 2000 rpm for 2 minutes to remove any air bubbles. All mAb-C samples were kept at the temperature of interest for 10 minutes for temperature equilibration and then fifteen 5 second acquisitions were collected for each sample. Dynamics 7.1.7 software was used to calculate the hydrodynamic diameter of the mAb-C samples. The solution viscosity of each buffer alone (placebo) was measured using an m-VROC viscometer (Rheosence, San Ramon, CA) at 25°C. Buffer viscosity correction was implemented in instrument software for calculation of the hydrodynamic diameter of mAb-C samples.

### Chemical cross-linking and SDS-PAGE analysis

An amine-reactive cross-linker, bis (sulfosuccinimidyl) 2,2,4,4, glutarate (BS^2^G), with a spacer arm length of 7.7 Å, was used to study reversible-self association of mAb-C. mAb-C was concentrated to 10 mg/mL and then dialyzed against 40 mM potassium phosphate buffer (pH 7.0) with and without 300 mM sodium sulfate, for 24 hours at 4°C. For the chemical cross-linking reaction, 10 mg/mL mAb-C in the presence and absence of sodium sulfate was incubated with increasing concentrations of BS^2^G ranging from 5- to 40-fold molar excess to mAb-C. The reaction mixtures were incubated at 4°C for 5 minutes and quenched by addition of 1 M tris hydrochloride. mAb-C from the reaction mixtures was combined with 4X lauryl dodecyl sulfate (LDS) buffer, iodoacetamide, and filtered deionized water to obtain a final sample volume of 20 μL containing 8 micrograms of protein, 50 mM iodoacetamide, and 1X LDS buffer. For reducing conditions, 100 mM DTT was added to the mAb-C-LDS solution. All samples were heated at 75°C for 5 minutes and then analyzed using a 3%–8% tris-acetate SDS-PAGE gel (Life Technologies, Grand Island, NY).

### Viscosity measurements

Prior to solution viscosity measurements, mAb-C stock was dialyzed against 40 mM potassium phosphate buffer (pH 7) containing either 300 mM NaCl or Na_2_SO_4_. Dialyzed samples were concentrated to 60 mg/mL and then diluted with dialysis buffer to make protein concentrations ranging from 5–60 mg/mL. Solution viscosities were measured at 4°C and 25°C with an m-VROC viscometer (Rheosence, San Ramon, CA). Samples at different concentrations of mAb-C were injected at a rate of 100 μL/min at a shear rate of 1420 1/s using a 1 mL glass syringe (Hamilton Co, Reno, NV). Triplicate viscosity measurements were recorded over a duration of 100 seconds. Viscosity values were obtained in the units of dynamic viscosity: mPa s.

### Lyophilization of mAb-C

Prior to freeze-drying, mAb-C samples were dialyzed against 20 mM potassium phosphate buffer (pH 7) with 10% (w/v) trehalose for 24 hours and then concentrated to 60 mg/mL. Half of the mAb-C samples were diluted in the same buffer to 5 mg/mL. The mAb-C samples at 5 and 60 mg/mL were filled into 3 mL FIOLAX® clear Type 1 glass vials (Schott North America, Elmsford, NY) with a fill volume of 500 μL. All the vials were partially stoppered by 2-leg, 13 mm siliconized rubber stoppers (Wheaton Industries Inc., Millville, NJ) The samples were then lyophilized using a LyoStar II lyophilizer (SP Scientific, Warminster, PA). Vials were subjected to an initial hold step at 5°C and then a freezing hold step at –40°C. During primary drying, the shelf temperature was maintained at –30°C for 400 minutes and then at –35°C for 800 minutes with the chamber pressure maintained at 100 mTorr. Secondary drying was done by ramping the shelf temperature at 0.1°C/min until reaching a final shelf temperature of 25°C for 60 minutes. Residual moisture levels of ∼0.5% (v/v) in the lyophilized mAb-C samples were determined using a Karl-Fischer titration unit according to vendor instructions (Mettler Toledo. LLC, Columbus, OH)

### Size exclusion chromatography (SEC)

Lyophilized mAb-C samples were reconstituted and diluted to 0.5 mg/mL with D_2_O-based 40 mM potassium phosphate buffer containing 300 mM sodium sulfate and 10% (w/v) trehalose. All mAb-C samples were centrifuged at 14000 g for 5 minutes prior to SEC analysis. For SEC experiments, a 7.8 cm × 30 cm TSK-Gel BioAssist G3SW_xL_ column (TOSOH Biosciences, King of Prussia, PA) column was used. Samples were injected onto the column at a flow rate of 0.7 mL/min with a Shimadzu high-performance liquid chromatography (HPLC) system equipped with a diode array detector using a 200 mM phosphate buffer (pH 6.8) as the mobile phase. The performance of the column and the HPLC system was monitored using gel filtration standards (Bio-Rad, Hercules, CA) at the beginning and end of the experiment. The chromatograms were analyzed by integrating the monomer peak area detected at 214 nm. Percent aggregation in post-lyophilization samples was measured relative to the total area of control samples (no lyophilization) at each protein concentration. To calculate the amount of insoluble aggregates, the total area of all the species (soluble aggregates, monomer, and fragment) in the chromatogram was calculated. The difference between the total peak areas of the sample and the control was used to quantify insoluble aggregates.

### Circular dichroism

Lyophilized mAb-C samples at 5 and 60 mg/mL were reconstituted to a concentration of 0.3 mg/mL with a D_2_O-based 40 mM potassium phosphate buffer containing 300 mM sodium sulfate and 10% (w/v) trehalose at pH 7.0. The protein concentration of each sample was measured by absorbance at 280 nm using a previously reported value of E_1 cm_ 0.1% of 1.54 mL mg^−1^cm^−1^.^11^ Circular dichroism (CD) experiments were conducted on a Chirascan Plus Circular Dichroism Spectrometer (Applied Photophysics Ltd., Leatherhead, UK) equipped with a Peltier temperature controller and a 4-position cuvette holder. Far-UV (UV) CD spectra of control (no lyophilization) and freeze-dried mAbC samples (0.3 mg/mL) were collected from 200 nm to 260 nm using 0.1 cm path length quartz cuvettes. Control mAb-C samples were prepared in H_2_O-based 40 mM potassium phosphate buffer containing 300 mM sodium sulfate and 10% (w/v) trehalose at pH 7.0. CD scans were collected at 10°C using a sampling time of 1 second and a bandwidth of 1 nm. Ellipticity values obtained from the instrument were then converted to molar ellipticity by dividing ellipticity by protein concentration (M) and cuvette path length (m).

### Deuterated reconstitution buffer preparation

Deuterium-based reconstitution/labeling buffer contained 20 mM potassium phosphate, 300 mM sodium sulfate and 10% (w/v) trehalose at pH 7. The amount of D_2_O in the reconstitution/labeling buffer was adjusted to 90 atom % by addition of H_2_O as described previously.^38^ The pH of the buffer was then adjusted to 7.0. The pH value of the solution was directly reported from the pH meter readout and does not include any correction for the deuterium isotope effect.^79^

### Hydrogen exchange mass spectrometry

Lyophilized vials containing 5 and 60 mg/mL mAb-C in 20 mM potassium phosphate and 10% (w/v) trehalose at pH 7.0, along with vials of the deuterium reconstitution/labeling buffer, were each equilibrated to 25°C on an Echotherm chilling/heating plate (Torrey Pines Scientific, Inc., Carlsbad, CA). A quench buffer containing 0.5 M tris(2-carboxyethyl)phosphine hydrochloride (TCEP), 4 M guanidine hydrochloride and 0.2 M sodium phosphate at pH 2.5 was equilibrated at 1°C. Deuterated reconstitution buffer (500 μL) was added to the lyophilized samples to yield a final protein concentration of 5 and 60 mg/mL and a final formulation composition of 40 mM potassium phosphate, 300 mM sodium sulfate, 10% (w/v) trehalose at pH 7.0 in D_2_O. After addition of deuterated reconstitution buffer to the vials containing lyophilized protein, the vials were gently swirled for ∼10 s until the freeze-dried cakes were reconstituted. The vials were held for an additional 60 s to allow foam to dissipate. The reconstituted mAb-C samples were then transferred to 2 mL microcentrifuge tubes and centrifuged at 14000 g for 1 minute to remove small amounts of insoluble aggregates present in the sample (see **Table 1**). Reconstituted, centrifuged samples of mAb-C were then incubated for 4 labeling times: 120, 1620, 10^4^ and 10^5^ s; the labeling period was started at the end of the dissolution step. After labeling, the exchange reaction was quenched by adding 20 μL of the exchange reaction mixture to 180 μL of quench buffer equilibrated at 1°C. Dilution with quench buffer was maintained for both protein concentration samples to keep the extent of back-exchange equal in the samples.

A LEAP H/DX PAL (LEAP Technologies, Carrboro, NC) was used to load quenched samples into the sample loop of the refrigerated column compartment containing 2 valves attached to the LC (Agilent 1200 series, Santa Clara, CA), an immobilized pepsin column, a peptide desalting trap, and a C18 column as described previously.^37,38^ Formic acid (0.1% v/v) was used for digestion and desalting. A mobile phase composed of water and acetonitrile, both containing 0.1% (v/v) formic acid, was use to elute the peptides. The temperature of the refrigerated compartment was maintained at 1°C during the course of the experiments. Different volumes of sample, 90 μL for the low, and 15 μL for the high concentrations were used so that the total amount of protein injected into the LC was similar: 45 μg for the low, and 90 μg for the high concentration samples. Three independent replicates for both protein concentrations were prepared and analyzed. To minimize peptide carryover in the immobilized pepsin column, the column was washed between samples following a cleaning procedure described previously^80^ except that 2 cycles of pepsin column wash were used after each injection. Deuteration was measured using a time-of-flight mass spectrometer (Agilent 6220, Santa Clara, CA) equipped with a standard electrospray ionization source operated in positive mode.

### HX-MS data processing and analysis

A combination of accurate mass measurements from time-of-flight MS and data obtained from collision induced dissociation with tandem MS on a linear quadrupole ion trap (LTQ-XL, Thermo-Scientific) was used to identify a total of 130 peptides. Identified peptides covered 94% of the primary sequence of the heavy and 93% of the light chain of mAb-C.

HDExaminer (Sierra Analytics, Modesto, CA) was used to analyze the hydrogen exchange data. Peptide mass spectra from all 3 replicates of both protein concentrations were manually curated after initial processing. Deuterium uptake plots with average deuterium uptake values and standard deviations for each peptide were generated by using an R script program, written in-house.

Statistical significance was determined following the method described by Houde et al. for replicate differential hydrogen exchange data. For our triplicate measurements, the 99th percentile of the standard deviations (*s*_99%_) was 0.28. For differential measurements, i.e., Δ*m*, the 99% confidence interval becomes $99\%\text{C}\text{I}=\sqrt{s_{99\%}^{2}+s_{99\%}^{2}}$. establishing a 99% confidence limit of ±0.4 Da for our dataset (see **Fig. S3**).^81^

Hydrogen exchange data for each peptide was mapped onto a homology model of mAb-C based on human IgG b12 (PDB ID: 1HZH)^82^ developed as described previously. ^45^ In some cases, peptides spanning the same region showed contradictory results: some indicated significant differences while others did not. At 99% confidence, false negatives are much more likely than false positives, peptides with significant differences “overruled” those that did not show significant differences. There were no cases where overlapping peptides indicated contradictory results (i.e., one peptide with Δm>0.4 and an overlapping peptide with Δm<−0.4). Pymol (Schrödinger LLC, Portland, OR) was used to display the HX-MS data.
