# Supplementary material for: Hydrogen exchange mass spectrometry reveals protein interfaces and distant dynamic coupling effects during the reversible self-association of an IgG1 monoclonal antibody
Source: MAbs. 2015 Apr 15;7(3):525–39. doi: 10.1080/19420862.2015.1029217 (PMC4622866; doi:10.1080/19420862.2015.1029217)

Supporting Information

Hydrogen exchange mass spectrometry reveals protein interfaces and distant dynamic coupling effects during the reversible self-association of an IgG1 monoclonal antibody

Jayant Arora[1], John M. Hickey[1], Ranajoy Majumdar[1], Reza Esfandiary[2], Steven M. Bishop[2], Hardeep S. Samra[2], C. Russell Middaugh[1], David D. Weis[3], David B. Volkin[1]

[1]Department of Pharmaceutical Chemistry, Macromolecule and Vaccine Stabilization Center, University of Kansas, Lawrence, Kansas, USA, 66047

[2]Department of Formulation Sciences, MedImmune LLC, Gaithersburg, Maryland, 20878

[3]Department of Chemistry and R.N. Adams Institute of Bioanalytical Chemistry, University of Kansas, Lawrence, Kansas, USA, 66045

Supplemental Table Legends

Supplemental Table S1: Location of pepsin generated peptide segments (from HX-MS analysis of mAb-C) in the mAb-C primary sequence and their corresponding unique peptide numbers.

Supplemental Figure Captions

Supplemental Figure S1: Pepsin peptide map of the (A) heavy chain (HC) and (B) light chain (LC) of mAb-C composed of 130 common peptide segments covering 94% of the HC primary sequence and 93% of the LC primary sequence.

Supplemental Figure S2: Deuterium uptake plots as measured by HX-MS of 130 peptide segments comparing hydrogen exchange kinetics between 5 and 60 mg/mL mAb-C samples. Domain location and peptide number of the segment are shown in parentheses. The error bars represent one standard deviation from three independent experiments. Blue trace represents mAb-C sample at 5 mg/mL and red trace represents mAb-C sample at 60 mg/mL protein concentration.

Supplemental Figure S3: Reproducibility of the HX-MS data represented by the distribution of standard deviations for the mass differences across all time points and segments of mAb-C from the triplicate experiments (N = 1040). The 99th percentile for standard deviations calculated from the dataset is 0.28 Da.

Supplemental Figure S4: Difference plot showing the relative mass change (Δm) of 35 selected mAb-C peptide segments comparing lyophilized vs. unlyophilized mAb-C samples at 6 mg/mL as measured by HX-MS. The differences in hydrogen exchange between lyophilized and unlyophilized samples of mAb-C at 6 mg/mL $\left[\Delta m(t) = m_{lyo}(t) - m_{unlyo}(t)\right]$ are plotted on the vertical axis. The positive bars represent peptides that show increased in hydrogen exchange after lyophilization/reconstitution. Negative bars represent peptides that show decreased hydrogen exchange upon lyophilization/reconstitution (refer to Figure 6 for more detailed information about difference plots).

Supplemental Figure S5: Difference plot showing relative mass change of 35 selected mAb-C peptide segments comparing lyophilized vs. unlyophilized mAb-C samples at 60 mg/mL as measured by HX-MS. The differences in hydrogen exchange between lyophilized and unlyophilized samples of mAb-C at 60 mg/mL $\left[\Delta m(t) = m_{lyo}(t) - m_{unlyo}(t)\right]$ are plotted on the vertical axis. The positive bars represent peptides that show increased in hydrogen exchange after lyophilization/reconstitution. Negative bars represent peptides that show decreased hydrogen exchange upon lyophilization/reconstitution (refer to Figure 6 for more detailed information about difference plots).

Supplemental Table S1

Peptide Number	Location
1	mAb-C Heavy 4-10 (VH)
2	mAb-C Heavy 4-17 (VH)
3	mAb-C Heavy 8-29 (VH)
4	mAb-C Heavy 11-22 (VH)
5	mAb-C Heavy 11-26 (VH)
6	mAb-C Heavy 27-35 (VH)
7	mAb-C Heavy 33-43 (VH)
8	mAb-C Heavy 33-44 (VH)
9	mAb-C Heavy 35-45 (VH)
10	mAb-C Heavy 35-59 (VH)
11	mAb-C Heavy 37-45 (VH)
12	mAb-C Heavy 37-48 (VH)
13	mAb-C Heavy 45-59 (VH)
14	mAb-C Heavy 46-59 (VH)
15	mAb-C Heavy 47-59 (VH)
16	mAb-C Heavy 49-60 (VH)
17	mAb-C Heavy 50-59 (VH)
18	mAb-C Heavy 50-60 (VH)
19	mAb-C Heavy 60-70 (VH)
20	mAb-C Heavy 60-71 (VH)
21	mAb-C Heavy 63-69 (VH)
22	mAb-C Heavy 71-78 (VH)
23	mAb-C Heavy 71-80 (VH)
24	mAb-C Heavy 81-86 (VH)
25	mAb-C Heavy 84-93 (VH)
26	mAb-C Heavy 87-94 (VH)
27	mAb-C Heavy 100-112 (VH)
28	mAb-C Heavy 101-112 (VH)
29	mAb-C Heavy 102-112 (VH)
30	mAb-C Heavy 113-122 (VH)
31	mAb-C Heavy 121-130 (CH1)
32	mAb-C Heavy 121-140 (CH1)
33	mAb-C Heavy 135-140 (CH1)
34	mAb-C Heavy 135-150 (CH1)
35	mAb-C Heavy 141-152 (CH1)
36	mAb-C Heavy 145-174 (CH1)
37	mAb-C Heavy 152-162 (CH1)
38	mAb-C Heavy 155-165 (CH1)
39	mAb-C Heavy 164-187 (CH1)

40	mAb-C Heavy 167-182 (CH1)
41	mAb-C Heavy 171-182 (CH1)
42	mAb-C Heavy 176-182 (CH1)
43	mAb-C Heavy 188-193 (CH1)
44	mAb-C Heavy 193-205 (CH1)
45	mAb-C Heavy 194-201 (CH1)
46	mAb-C Heavy 199-211 (CH1)
47	mAb-C Heavy 219-227 (CH1)
48	mAb-C Heavy 229-254 (CH2)
49	mAb-C Heavy 233-252 (CH2)
50	mAb-C Heavy 243-248 (CH2)
51	mAb-C Heavy 246-256 (CH2)
52	mAb-C Heavy 249-260 (CH2)
53	mAb-C Heavy 251-260 (CH2)
54	mAb-C Heavy 252-259 (CH2)
55	mAb-C Heavy 261-268 (CH2)
56	mAb-C Heavy 261-269 (CH2)
57	mAb-C Heavy 261-271 (CH2)
58	mAb-C Heavy 270-285 (CH2)
59	mAb-C Heavy 271-285 (CH2)
60	mAb-C Heavy 273-285 (CH2)
61	mAb-C Heavy 282-284 (CH2)
62	mAb-C Heavy 285-298 (CH2)
63	mAb-C Heavy 308-314 (CH2)
64	mAb-C Heavy 311-325 (CH2)
65	mAb-C Heavy 315-341 (CH2)
66	mAb-C Heavy 320-334 (CH2)
67	mAb-C Heavy 342-356 (CH2)
68	mAb-C Heavy 344-356 (CH3)
69	mAb-C Heavy 357-366 (CH3)
70	mAb-C Heavy 365-372 (CH3)
71	mAb-C Heavy 365-374 (CH3)
72	mAb-C Heavy 377-388 (CH3)
73	mAb-C Heavy 385-398 (CH3)
74	mAb-C Heavy 385-406 (CH3)
75	mAb-C Heavy 385-412 (CH3)
76	mAb-C Heavy 389-398 (CH3)
77	mAb-C Heavy 389-406 (CH3)
78	mAb-C Heavy 389-412 (CH3)
79	mAb-C Heavy 390-406 (CH3)
80	mAb-C Heavy 399-406 (CH3)
81	mAb-C Heavy 399-412 (CH3)
82	mAb-C Heavy 407-412 (CH3)

83	mAb-C Heavy 413-418 (CH3)
84	mAb-C Heavy 417-428 (CH3)
85	mAb-C Heavy 419-431 (CH3)
86	mAb-C Heavy 420-436 (CH3)
87	mAb-C Heavy 431-454 (CH3)
88	mAb-C Heavy 432-438 (CH3)
89	mAb-C Heavy 432-454 (CH3)
90	mAb-C Heavy 434-454 (CH3)
91	mAb-C Heavy 436-454 (CH3)
92	mAb-C Heavy 437-454 (CH3)
93	mAb-C Light 1-10 (VL)
94	mAb-C Light 1-12 (VL)
95	mAb-C Light 4-10 (VL)
96	mAb-C Light 11-22 (VL)
97	mAb-C Light 13-32 (VL)
98	mAb-C Light 33-49 (VL)
99	mAb-C Light 35-48 (VL)
100	mAb-C Light 36-54 (VL)
101	mAb-C Light 47-62 (VL)
102	mAb-C Light 48-70 (VL)
103	mAb-C Light 49-70 (VL)
104	mAb-C Light 49-71 (VL)
105	mAb-C Light 50-71 (VL)
106	mAb-C Light 55-71 (VL)
107	mAb-C Light 72-82 (VL)
108	mAb-C Light 74-82 (VL)
109	mAb-C Light 75-82 (VL)
110	mAb-C Light 80-83 (VL)
111	mAb-C Light 88-104 (VL)
112	mAb-C Light 102-115 (VL)
113	mAb-C Light 105-116 (VL)
114	mAb-C Light 106-115 (VL)
115	mAb-C Light 116-131 (VL)
116	mAb-C Light 117-130 (CL)
117	mAb-C Light 117-133 (CL)
118	mAb-C Light 123-133 (CL)
119	mAb-C Light 124-133 (CL)
120	mAb-C Light 132-146 (CL)
121	mAb-C Light 136-143 (CL)
122	mAb-C Light 149-172 (CL)
123	mAb-C Light 155-172 (CL)
124	mAb-C Light 160-172 (CL)
125	mAb-C Light 162-172 (CL)

126	mAb-C Light 162-178 (CL)
127	mAb-C Light 173-179 (CL)
128	mAb-C Light 179-184 (CL)
129	mAb-C Light 180-194 (CL)
130	mAb-C Light 203-214 (CL)

Supplemental Figure S1

A

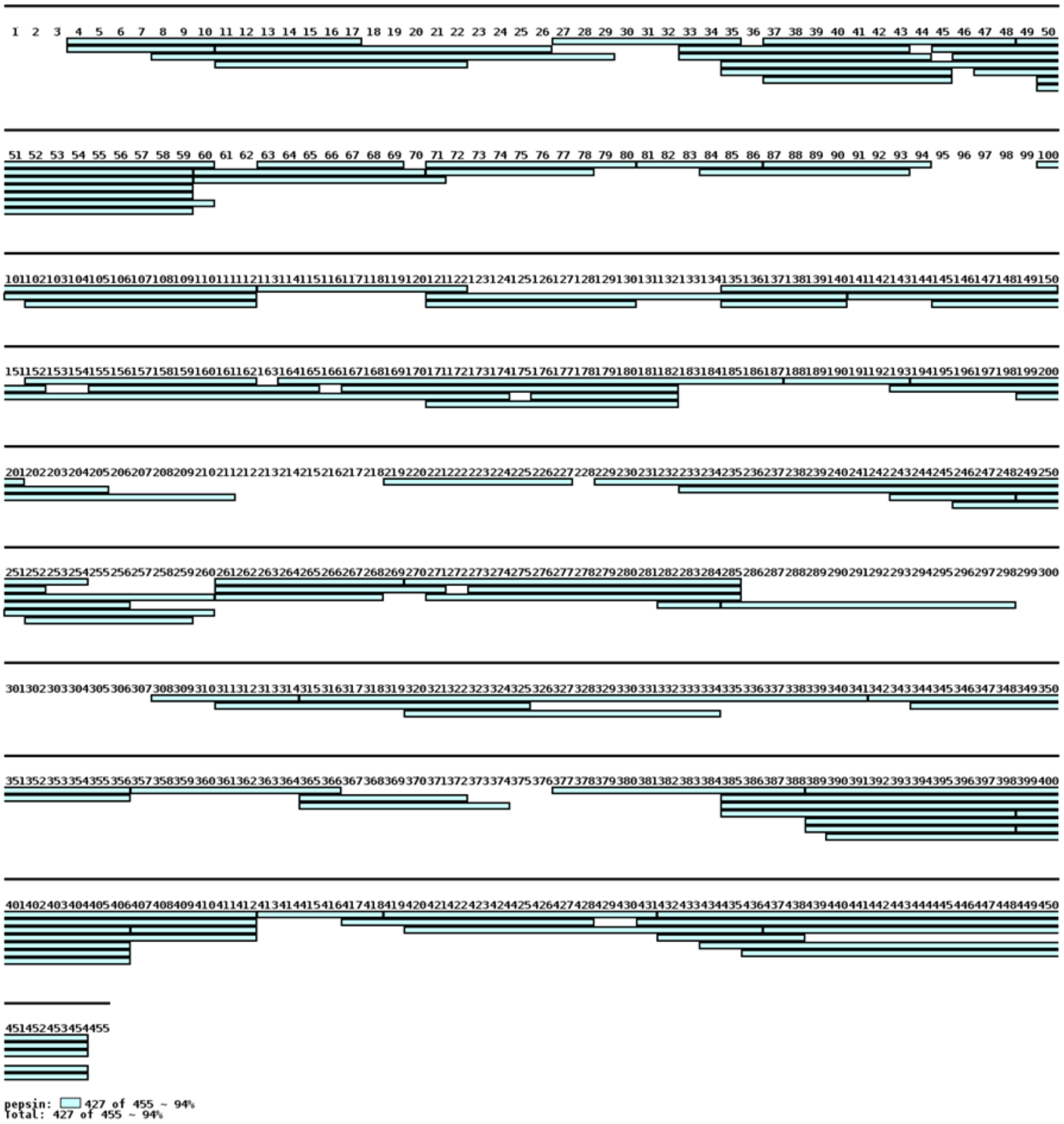

B

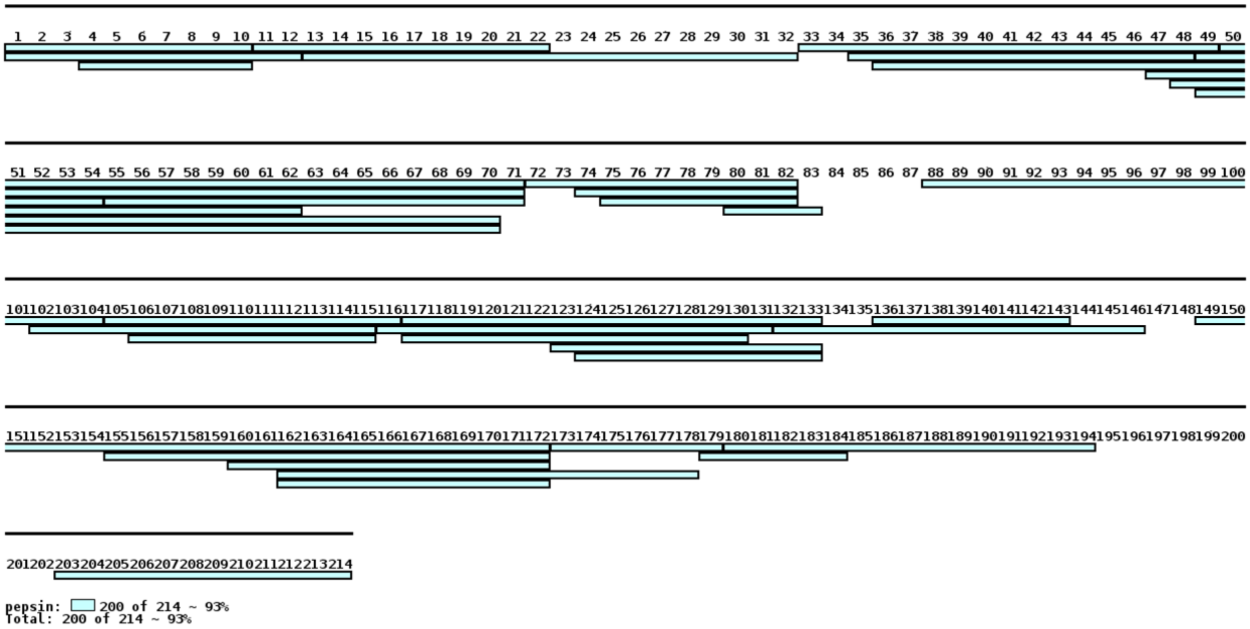

Supplemental Figure S2

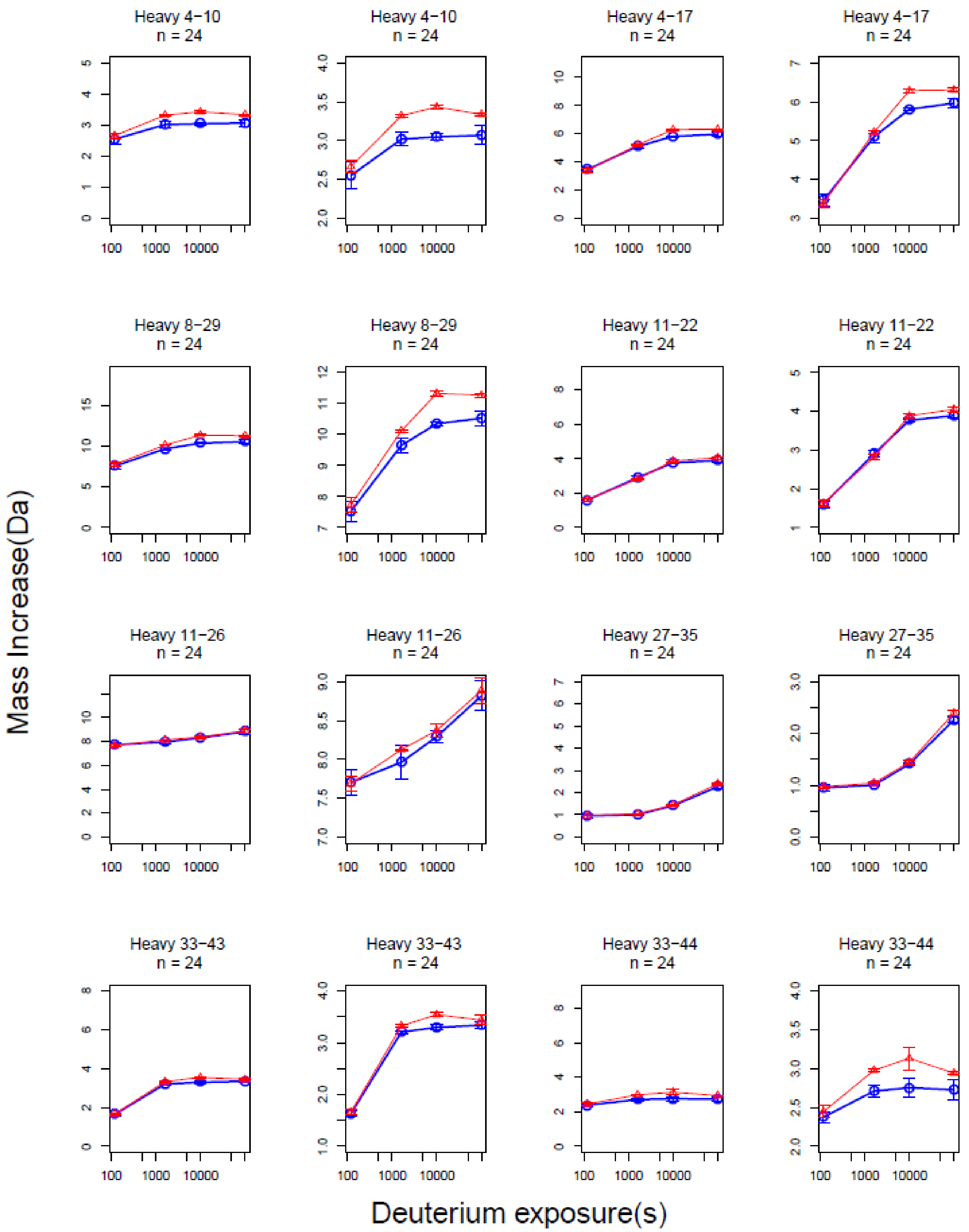
Heavy 4−10
n = 24
Heavy 4−10
n = 24
Heavy 4−17
n = 24
Heavy 4−17
n = 24
Heavy 8−29
n = 24
Heavy 8−29
n = 24
Heavy 11−22
n = 24
Heavy 11−22
n = 24
Heavy 11−26
n = 24
Heavy 11−26
n = 24
Heavy 27−35
n = 24
Heavy 27−35
n = 24
Heavy 33−43
n = 24
Heavy 33−43
n = 24
Heavy 33−44
n = 24
Heavy 33−44
n = 24
100
1000
10000
Mass Increase(Da)
Deuterium exposure(s)

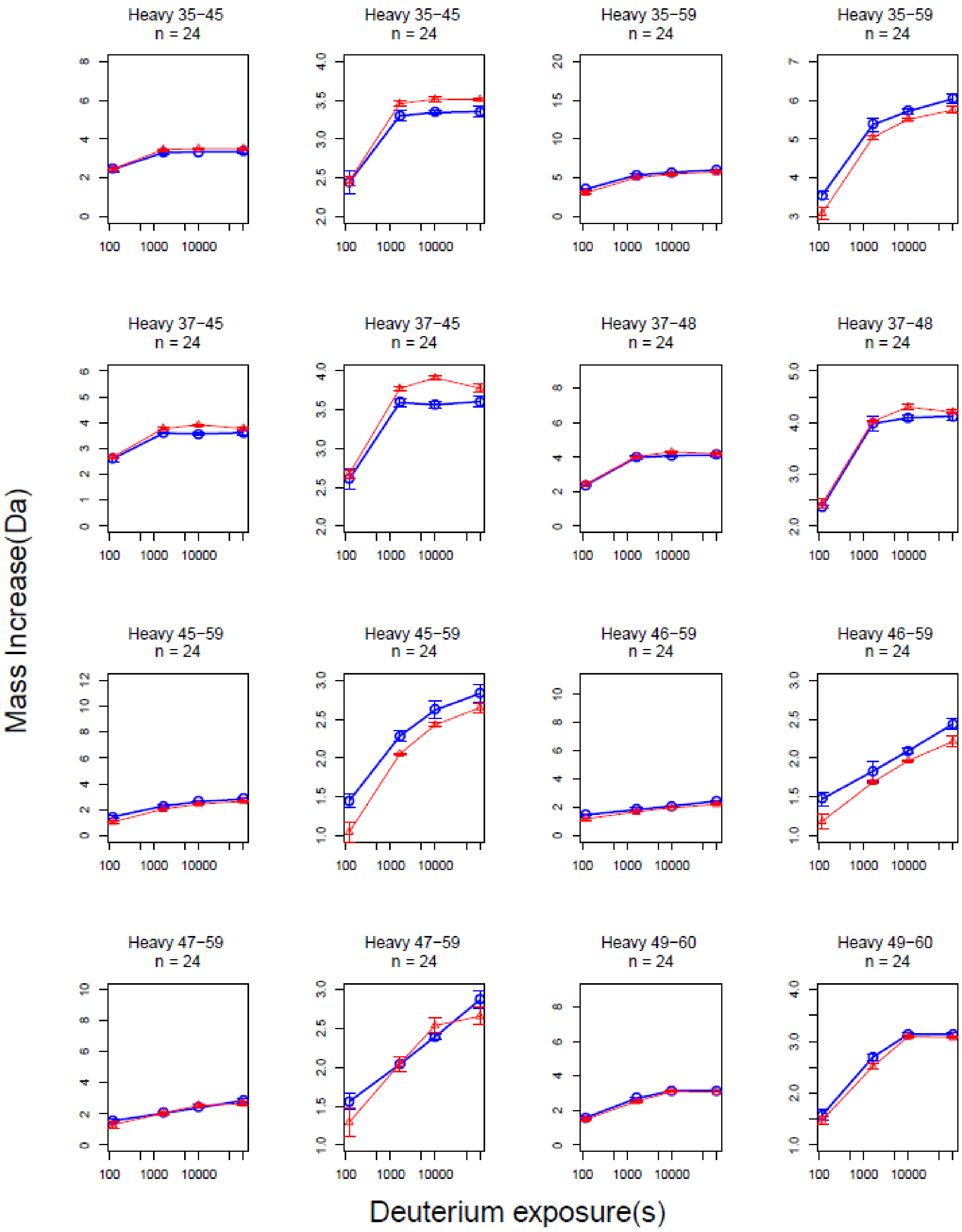
Heavy 35−45
n = 24
Heavy 35−59
Heavy 37−45
Heavy 37−48
Heavy 45−59
Heavy 46−59
Heavy 47−59
Heavy 49−60
100
1000
10000
Mass Increase(Da)
Deuterium exposure(s)

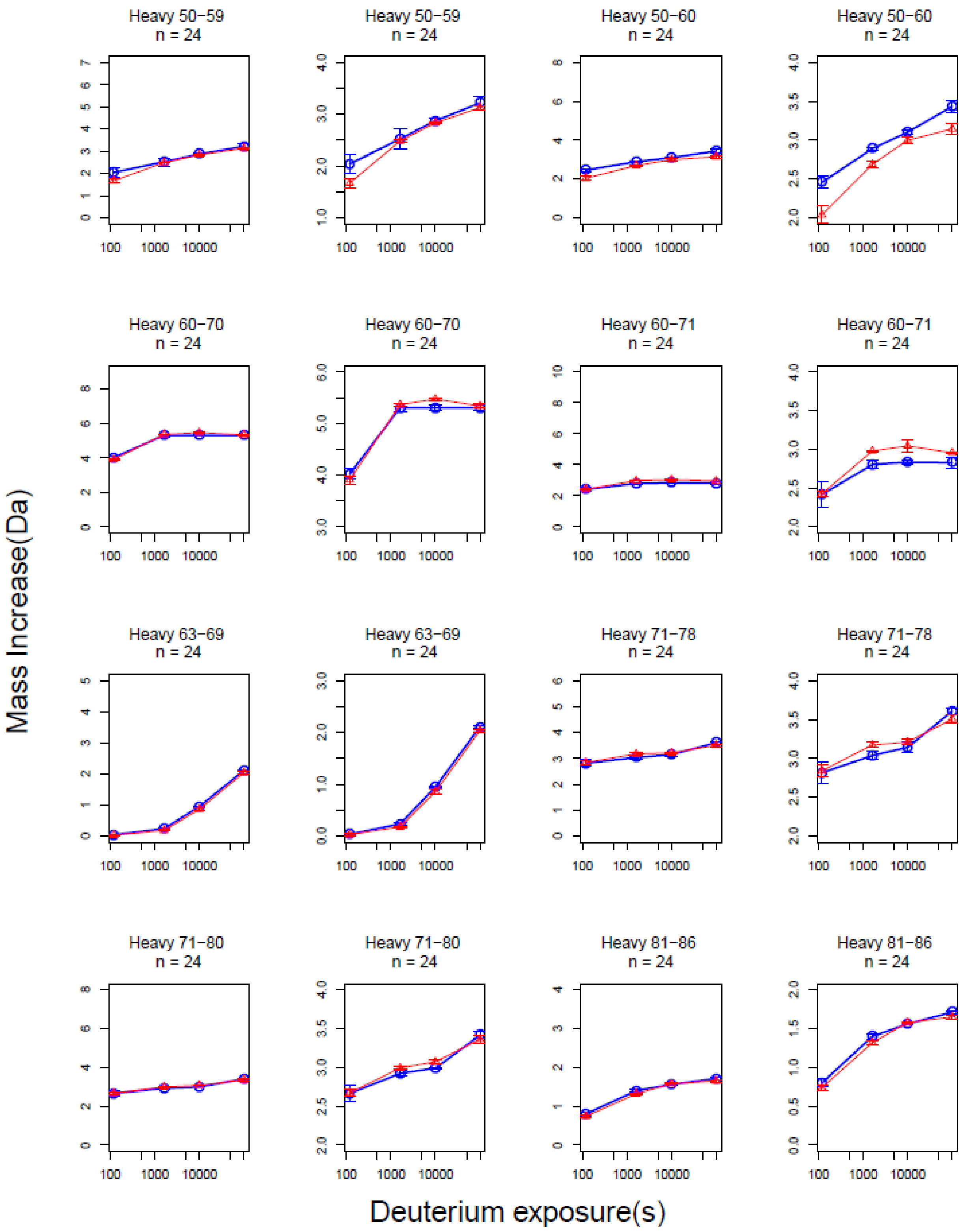
Heavy 50−59
n = 24
Heavy 50−59
n = 24
Heavy 50−60
n = 24
Heavy 50−60
n = 24
Heavy 60−70
n = 24
Heavy 60−70
n = 24
Heavy 60−71
n = 24
Heavy 60−71
n = 24
Heavy 63−69
n = 24
Heavy 63−69
n = 24
Heavy 71−78
n = 24
Heavy 71−78
n = 24
Heavy 71−80
n = 24
Heavy 71−80
n = 24
Heavy 81−86
n = 24
Heavy 81−86
n = 24
Mass Increase(Da)
Deuterium exposure(s)
100
1000
10000

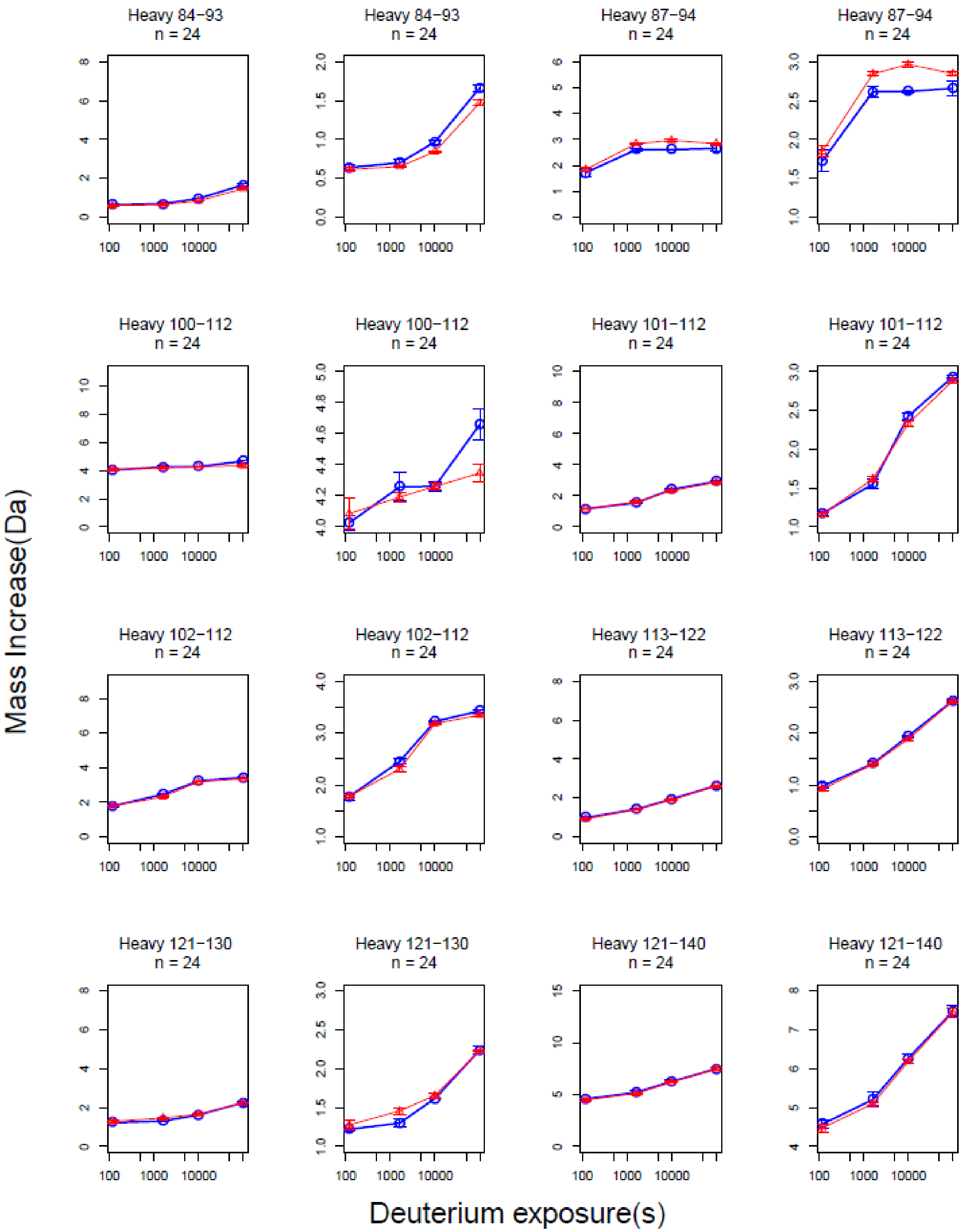

Heavy 84−93
n = 24
Heavy 84−93
n = 24
Heavy 87−94
n = 24
Heavy 87−94
n = 24
Heavy 100−112
n = 24
Heavy 100−112
n = 24
Heavy 101−112
n = 24
Heavy 101−112
n = 24
Heavy 102−112
n = 24
Heavy 102−112
n = 24
Heavy 113−122
n = 24
Heavy 113−122
n = 24
Heavy 121−130
n = 24
Heavy 121−130
n = 24
Heavy 121−140
n = 24
Heavy 121−140
n = 24
100
1000
10000
Mass Increase(Da)
Deuterium exposure(s)

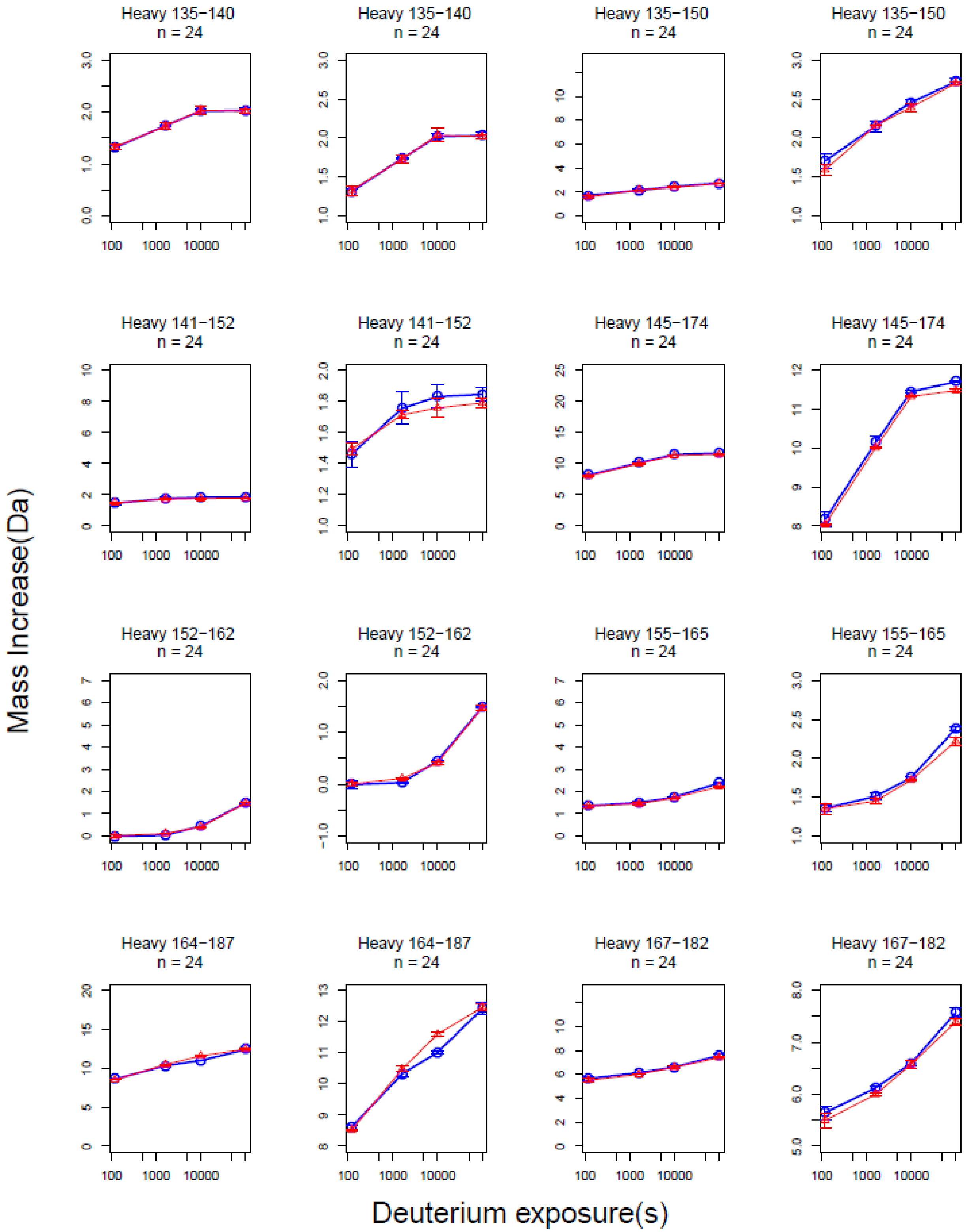
Heavy 135−140
n = 24
Heavy 135−140
n = 24
Heavy 135−150
n = 24
Heavy 135−150
n = 24
Heavy 141−152
n = 24
Heavy 141−152
n = 24
Heavy 145−174
n = 24
Heavy 145−174
n = 24
Heavy 152−162
n = 24
Heavy 152−162
n = 24
Heavy 155−165
n = 24
Heavy 155−165
n = 24
Heavy 164−187
n = 24
Heavy 164−187
n = 24
Heavy 167−182
n = 24
Heavy 167−182
n = 24
Mass Increase(Da)
Deuterium exposure(s)

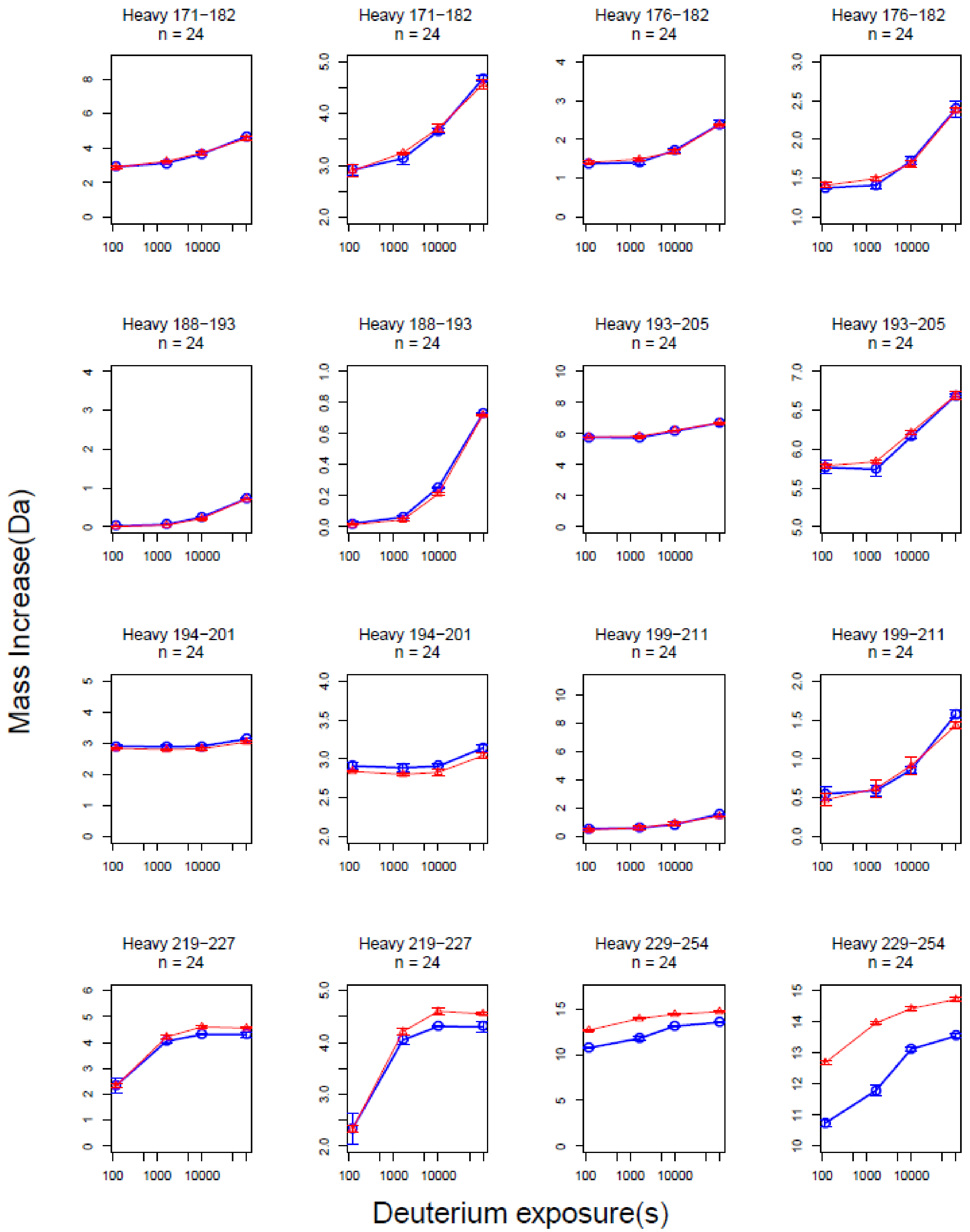
Heavy 171−182
n = 24
Heavy 171−182
n = 24
Heavy 176−182
n = 24
Heavy 176−182
n = 24
Heavy 188−193
n = 24
Heavy 188−193
n = 24
Heavy 193−205
n = 24
Heavy 193−205
n = 24
Heavy 194−201
n = 24
Heavy 194−201
n = 24
Heavy 199−211
n = 24
Heavy 199−211
n = 24
Heavy 219−227
n = 24
Heavy 219−227
n = 24
Heavy 229−254
n = 24
Heavy 229−254
n = 24
Mass Increase(Da)
Deuterium exposure(s)

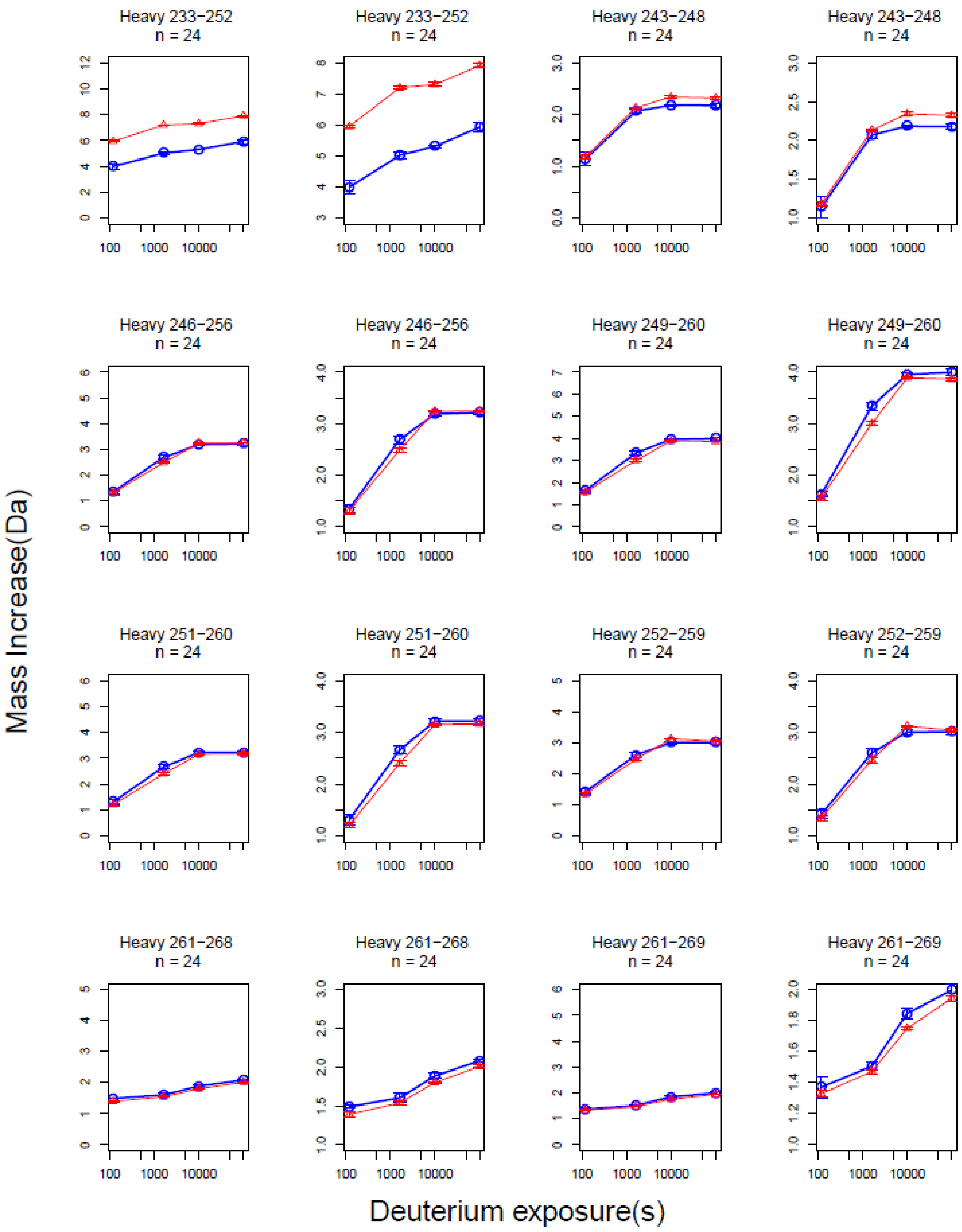
Heavy 233−252
n = 24
Heavy 233−252
n = 24
Heavy 243−248
n = 24
Heavy 243−248
n = 24
Heavy 246−256
n = 24
Heavy 246−256
n = 24
Heavy 249−260
n = 24
Heavy 249−260
n = 24
Heavy 251−260
n = 24
Heavy 251−260
n = 24
Heavy 252−259
n = 24
Heavy 252−259
n = 24
Heavy 261−268
n = 24
Heavy 261−268
n = 24
Heavy 261−269
n = 24
Heavy 261−269
n = 24
Mass Increase(Da)
Deuterium exposure(s)

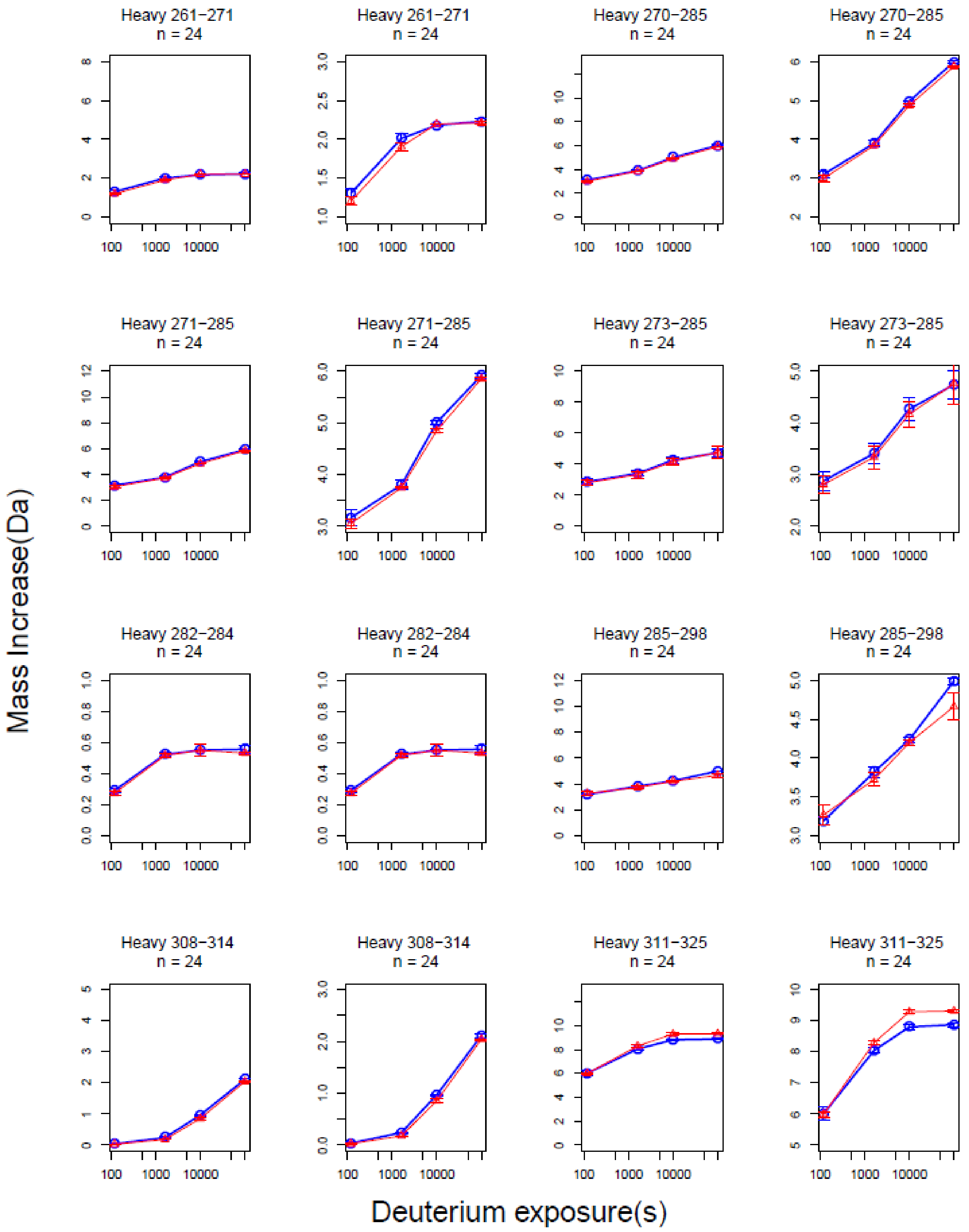
Heavy 261−271
n = 24
Heavy 261−271
n = 24
Heavy 270−285
n = 24
Heavy 270−285
n = 24
Heavy 271−285
n = 24
Heavy 271−285
n = 24
Heavy 273−285
n = 24
Heavy 273−285
n = 24
Heavy 282−284
n = 24
Heavy 282−284
n = 24
Heavy 285−298
n = 24
Heavy 285−298
n = 24
Heavy 308−314
n = 24
Heavy 308−314
n = 24
Heavy 311−325
n = 24
Heavy 311−325
n = 24
Mass Increase(Da)
Deuterium exposure(s)

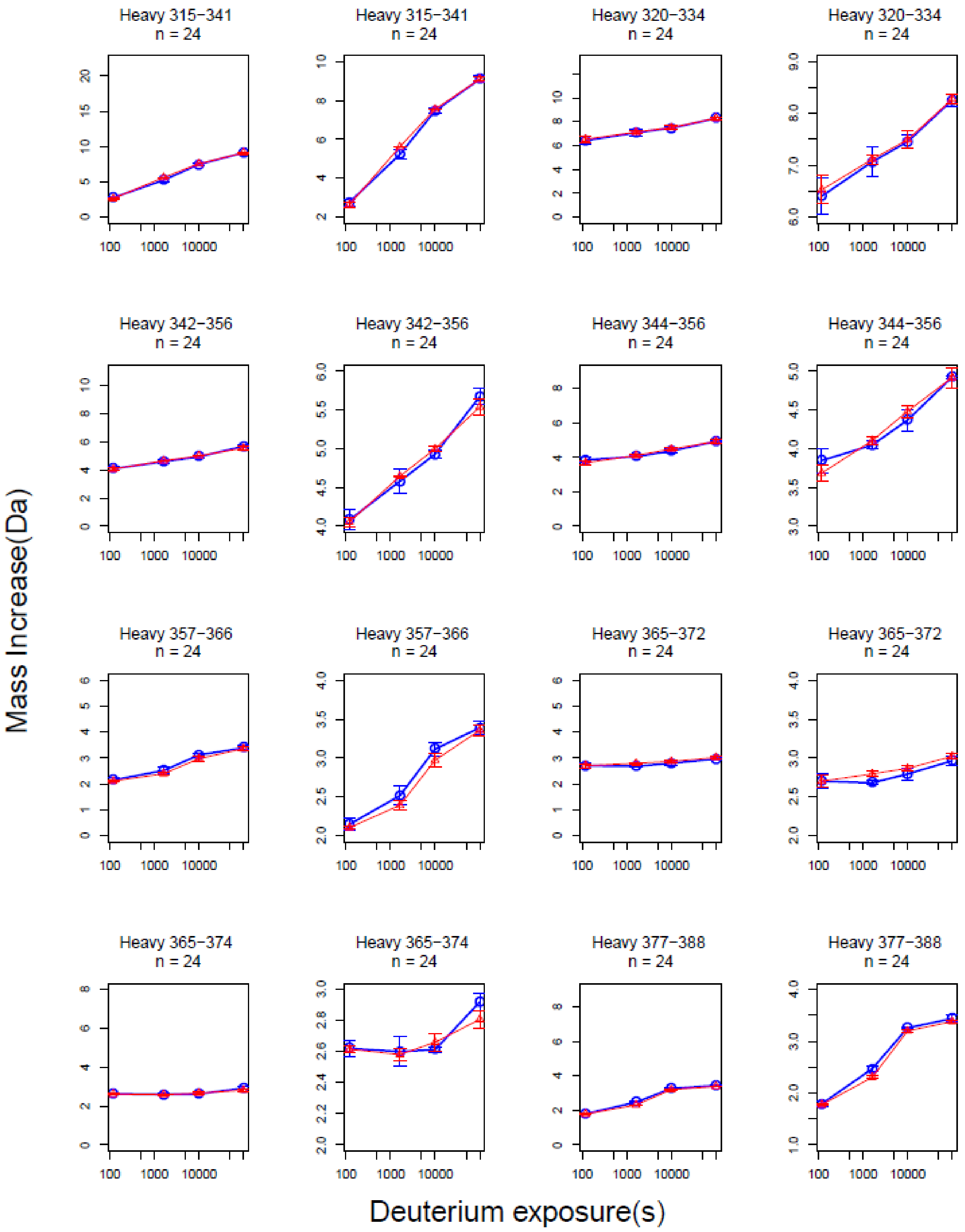
Heavy 315−341
n = 24
Heavy 315−341
n = 24
Heavy 320−334
n = 24
Heavy 320−334
n = 24
Heavy 342−356
n = 24
Heavy 342−356
n = 24
Heavy 344−356
n = 24
Heavy 344−356
n = 24
Heavy 357−366
n = 24
Heavy 357−366
n = 24
Heavy 365−372
n = 24
Heavy 365−372
n = 24
Heavy 365−374
n = 24
Heavy 365−374
n = 24
Heavy 377−388
n = 24
Heavy 377−388
n = 24
Mass Increase(Da)
Deuterium exposure(s)
100
1000
10000

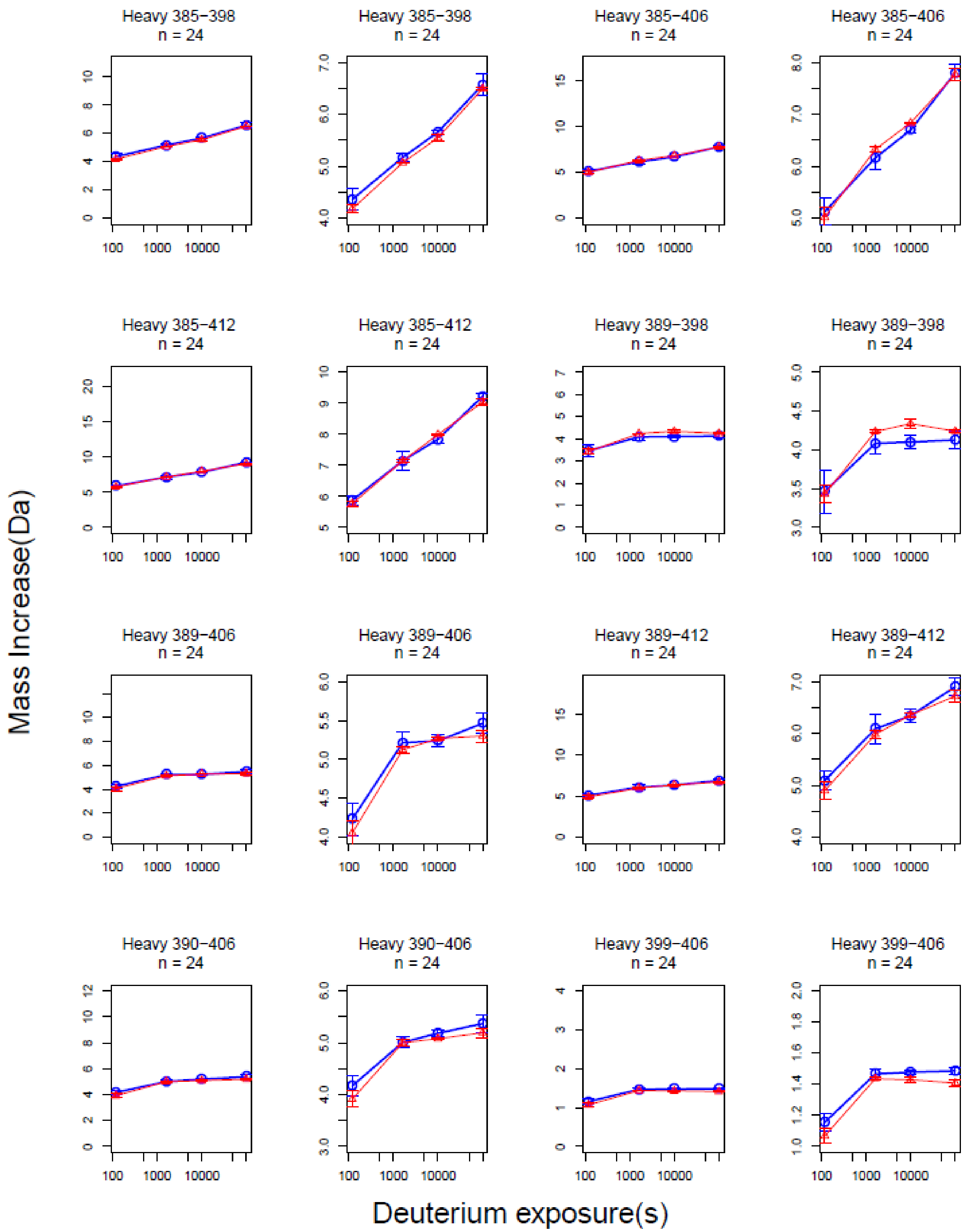
Heavy 385−398
n = 24
Heavy 385−398
n = 24
Heavy 385−406
n = 24
Heavy 385−406
n = 24
Heavy 385−412
n = 24
Heavy 385−412
n = 24
Heavy 389−398
n = 24
Heavy 389−398
n = 24
Heavy 389−406
n = 24
Heavy 389−406
n = 24
Heavy 389−412
n = 24
Heavy 389−412
n = 24
Heavy 390−406
n = 24
Heavy 390−406
n = 24
Heavy 399−406
n = 24
Heavy 399−406
n = 24
Mass Increase(Da)
Deuterium exposure(s)

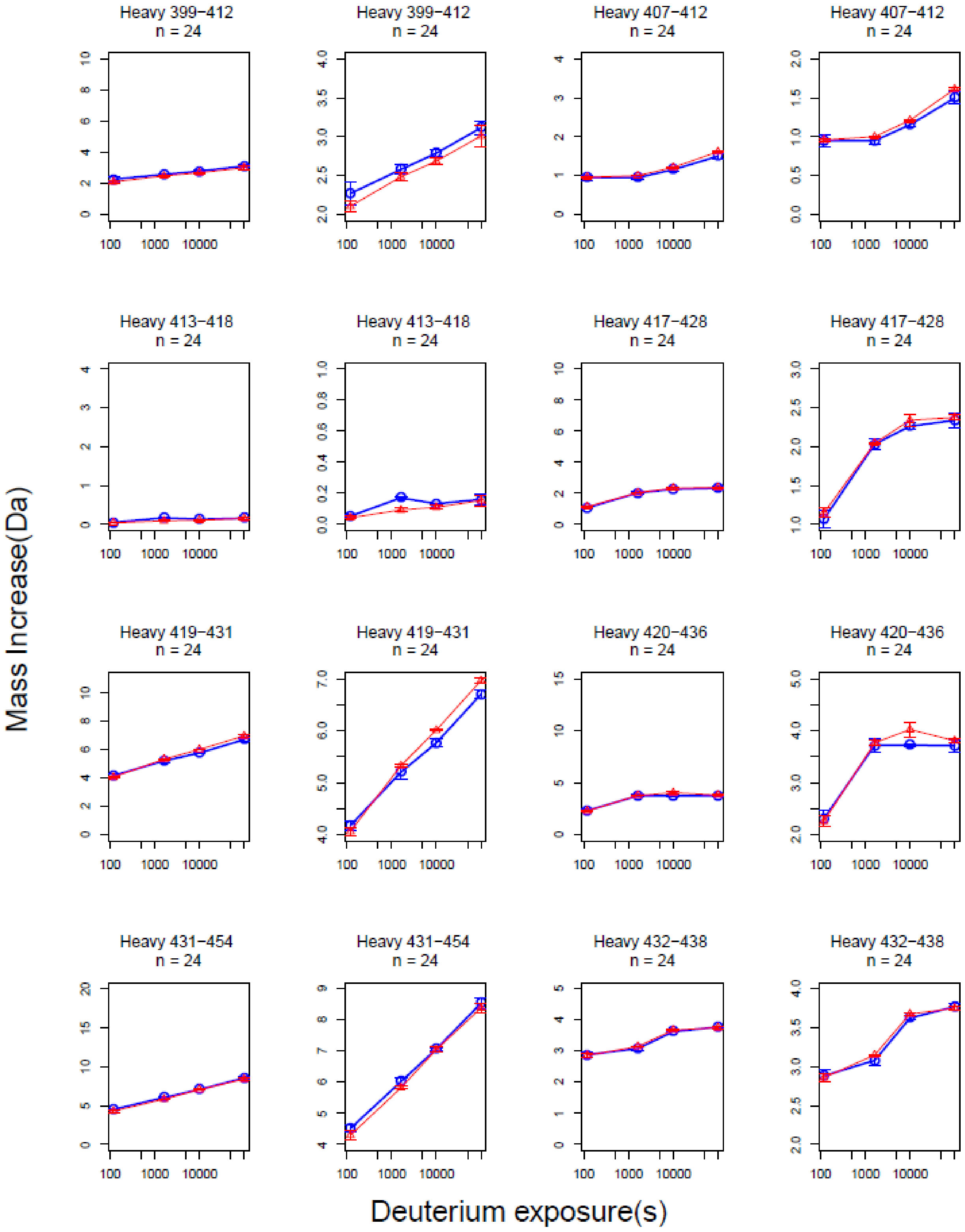
Heavy 399−412
n = 24
Heavy 399−412
n = 24
Heavy 407−412
n = 24
Heavy 407−412
n = 24
Heavy 413−418
n = 24
Heavy 413−418
n = 24
Heavy 417−428
n = 24
Heavy 417−428
n = 24
Heavy 419−431
n = 24
Heavy 419−431
n = 24
Heavy 420−436
n = 24
Heavy 420−436
n = 24
Heavy 431−454
n = 24
Heavy 431−454
n = 24
Heavy 432−438
n = 24
Heavy 432−438
n = 24
Mass Increase(Da)
Deuterium exposure(s)
100
1000
10000

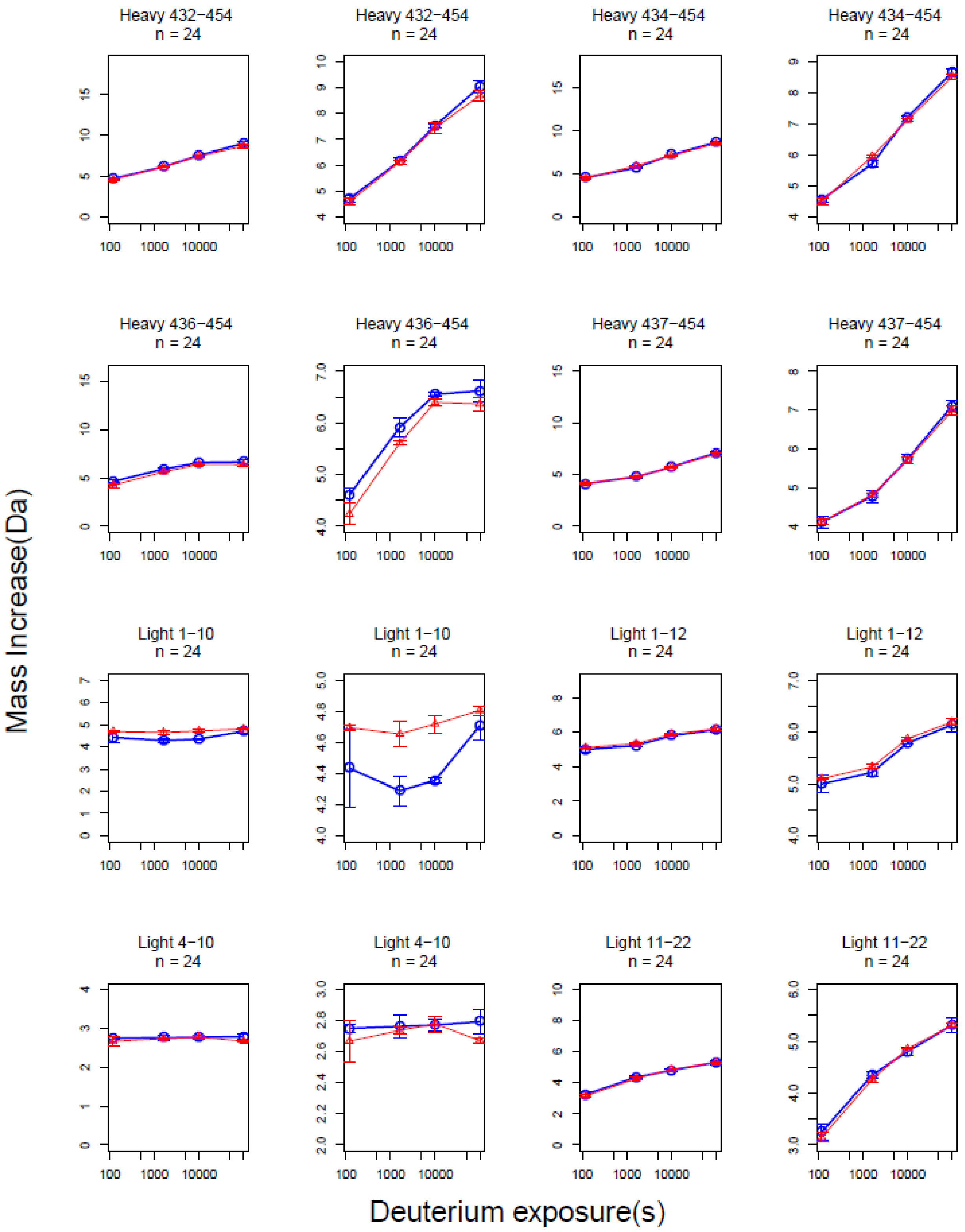

Heavy 432−454
n = 24
Heavy 432−454
n = 24
Heavy 434−454
n = 24
Heavy 434−454
n = 24
Heavy 436−454
n = 24
Heavy 436−454
n = 24
Heavy 437−454
n = 24
Heavy 437−454
n = 24
Light 1−10
n = 24
Light 1−10
n = 24
Light 1−12
n = 24
Light 1−12
n = 24
Light 4−10
n = 24
Light 4−10
n = 24
Light 11−22
n = 24
Light 11−22
n = 24
Mass Increase(Da)
Deuterium exposure(s)

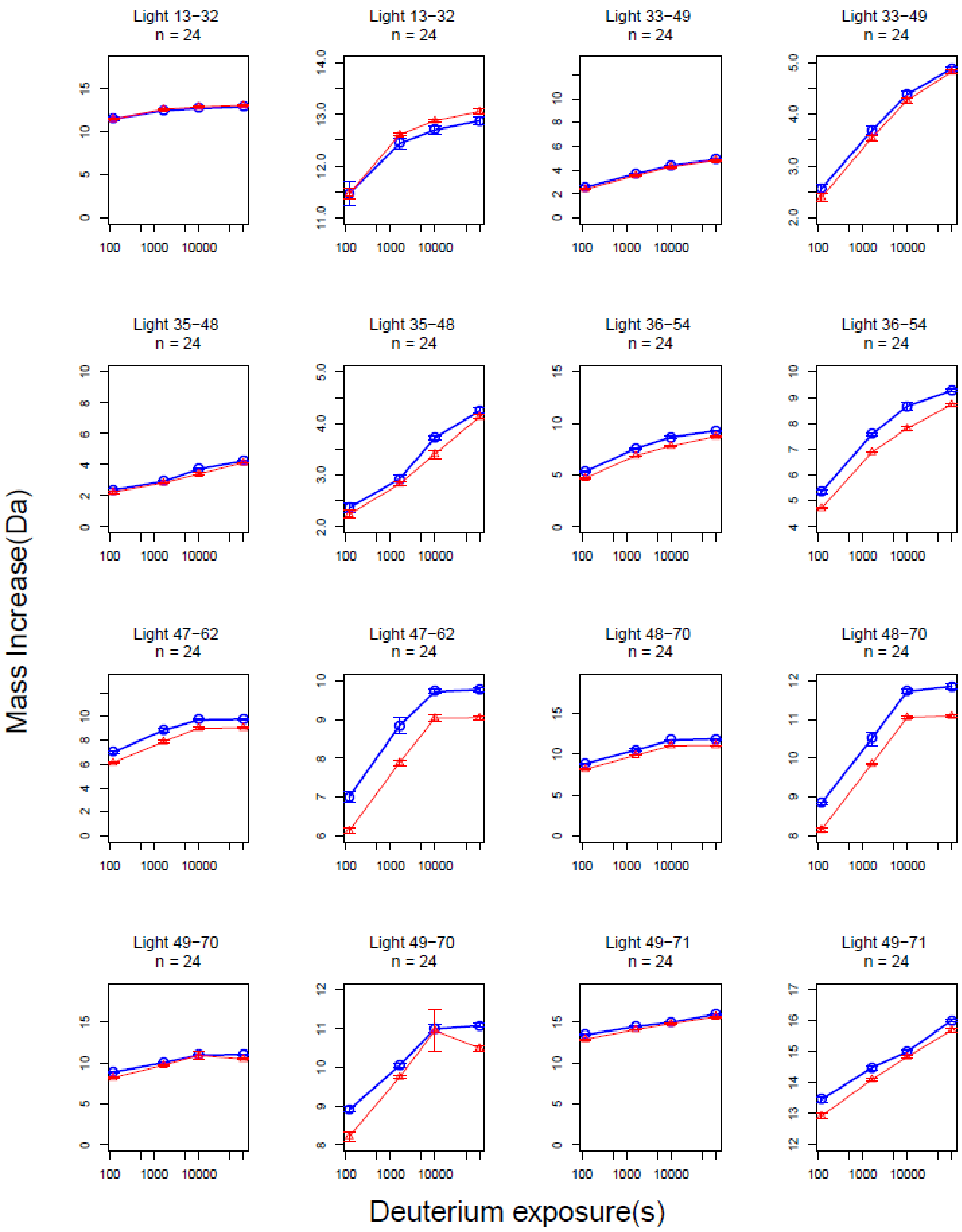
Light 13−32
n = 24
Light 13−32
n = 24
Light 33−49
n = 24
Light 33−49
n = 24
Light 35−48
n = 24
Light 35−48
n = 24
Light 36−54
n = 24
Light 36−54
n = 24
Light 47−62
n = 24
Light 47−62
n = 24
Light 48−70
n = 24
Light 48−70
n = 24
Light 49−70
n = 24
Light 49−70
n = 24
Light 49−71
n = 24
Light 49−71
n = 24
Mass Increase(Da)
Deuterium exposure(s)

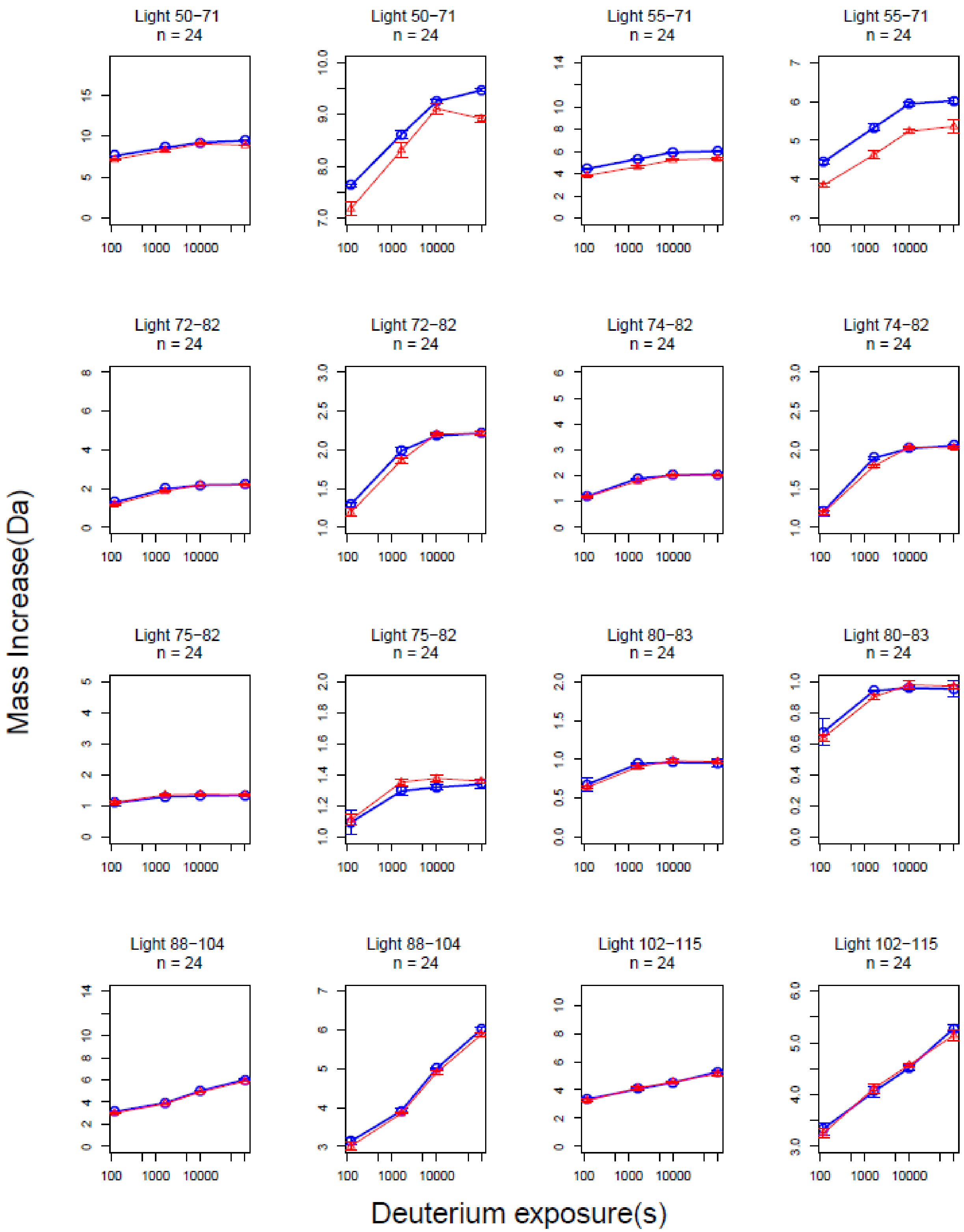
Light 50−71
n = 24
Light 50−71
n = 24
Light 55−71
n = 24
Light 55−71
n = 24
Light 72−82
n = 24
Light 72−82
n = 24
Light 74−82
n = 24
Light 74−82
n = 24
Light 75−82
n = 24
Light 75−82
n = 24
Light 80−83
n = 24
Light 80−83
n = 24
Light 88−104
n = 24
Light 88−104
n = 24
Light 102−115
n = 24
Light 102−115
n = 24
Mass Increase(Da)
Deuterium exposure(s)
100
1000
10000

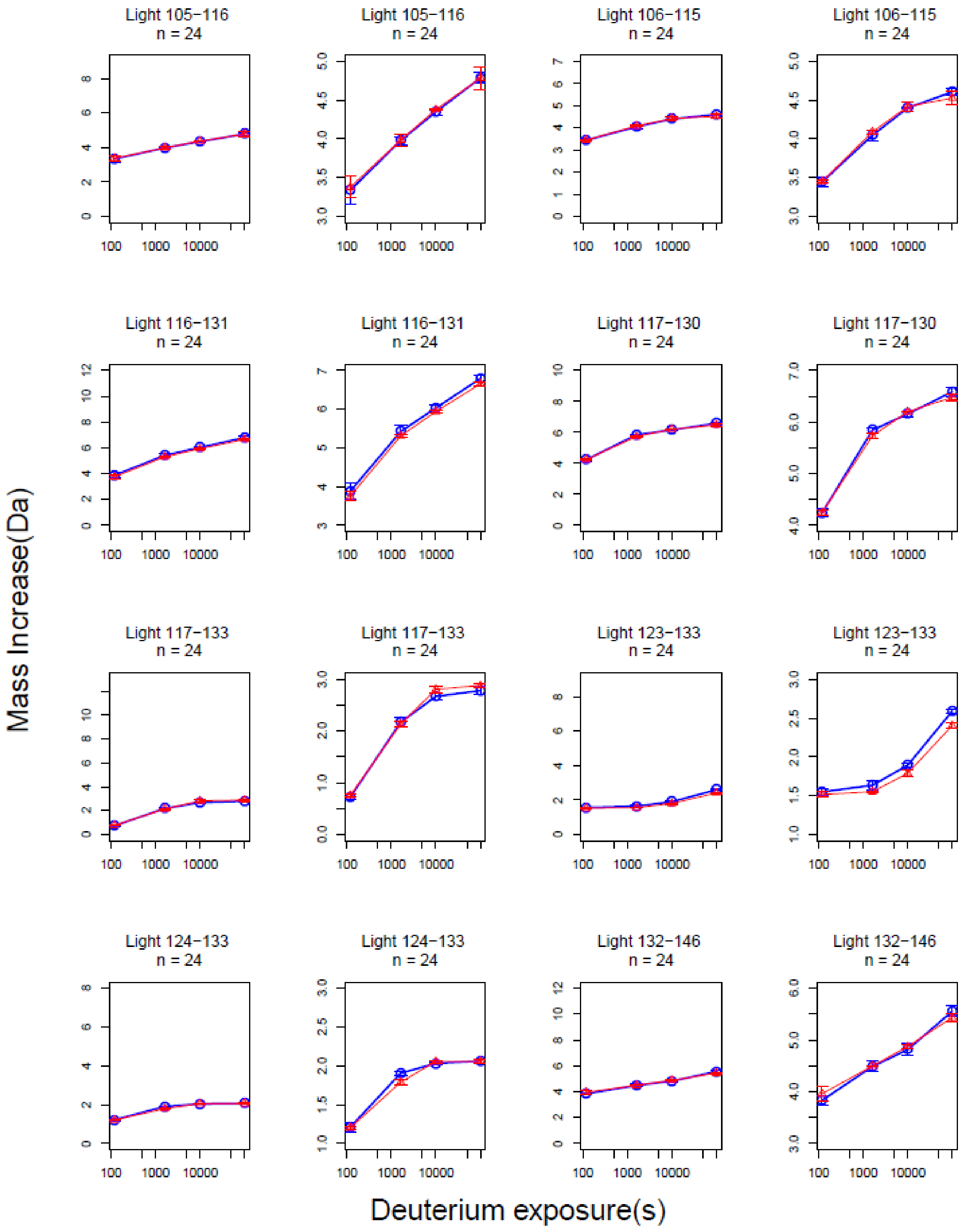
Light 105−116
n = 24
Light 105−116
n = 24
Light 106−115
n = 24
Light 106−115
n = 24
Light 116−131
n = 24
Light 116−131
n = 24
Light 117−130
n = 24
Light 117−130
n = 24
Light 117−133
n = 24
Light 117−133
n = 24
Light 123−133
n = 24
Light 123−133
n = 24
Light 124−133
n = 24
Light 124−133
n = 24
Light 132−146
n = 24
Light 132−146
n = 24
Mass Increase(Da)
Deuterium exposure(s)

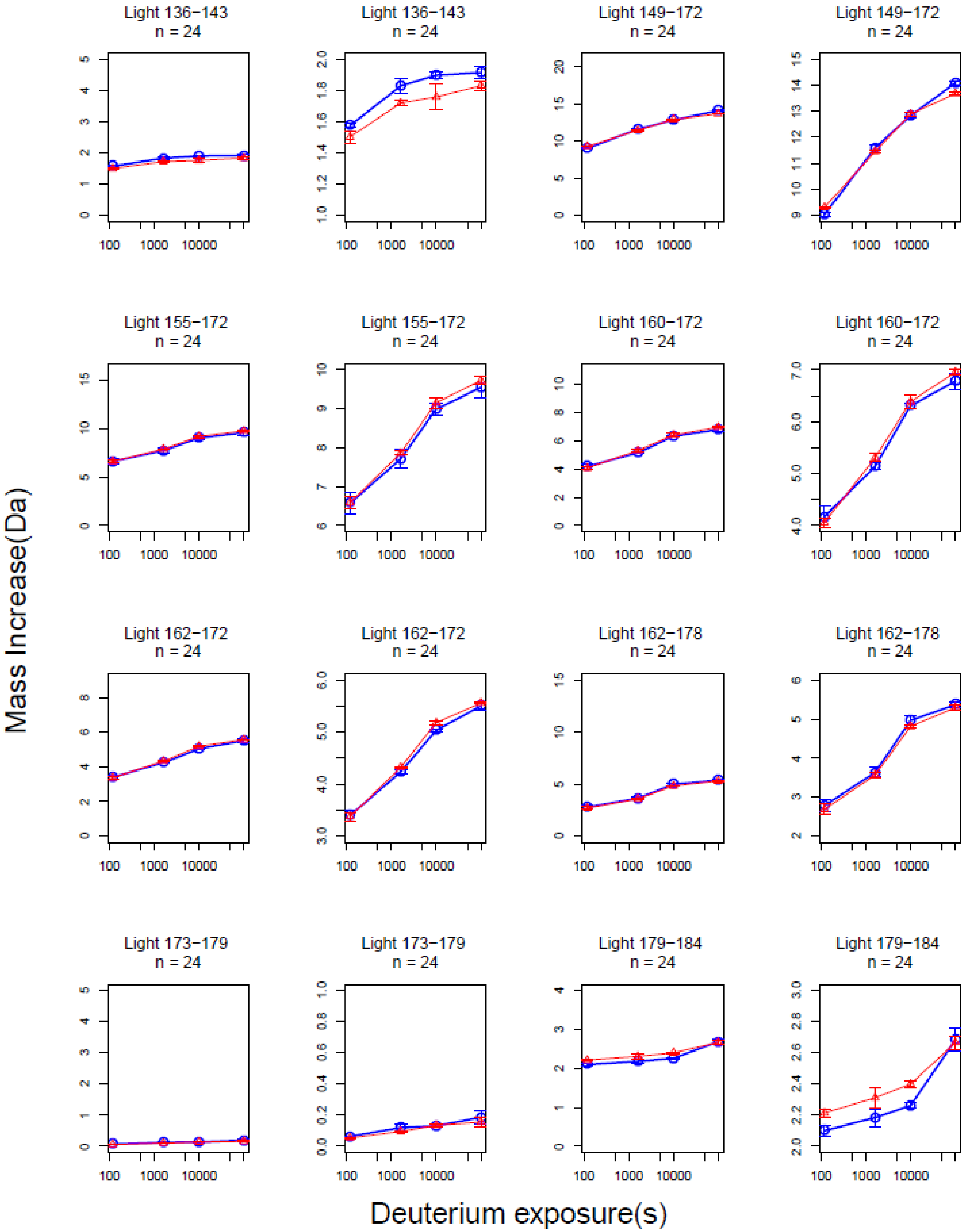
Light 136−143
n = 24
Light 136−143
n = 24
Light 149−172
n = 24
Light 149−172
n = 24
Light 155−172
n = 24
Light 155−172
n = 24
Light 160−172
n = 24
Light 160−172
n = 24
Light 162−172
n = 24
Light 162−172
n = 24
Light 162−178
n = 24
Light 162−178
n = 24
Light 173−179
n = 24
Light 173−179
n = 24
Light 179−184
n = 24
Light 179−184
n = 24
100 1000 10000
Mass Increase(Da)
Deuterium exposure(s)

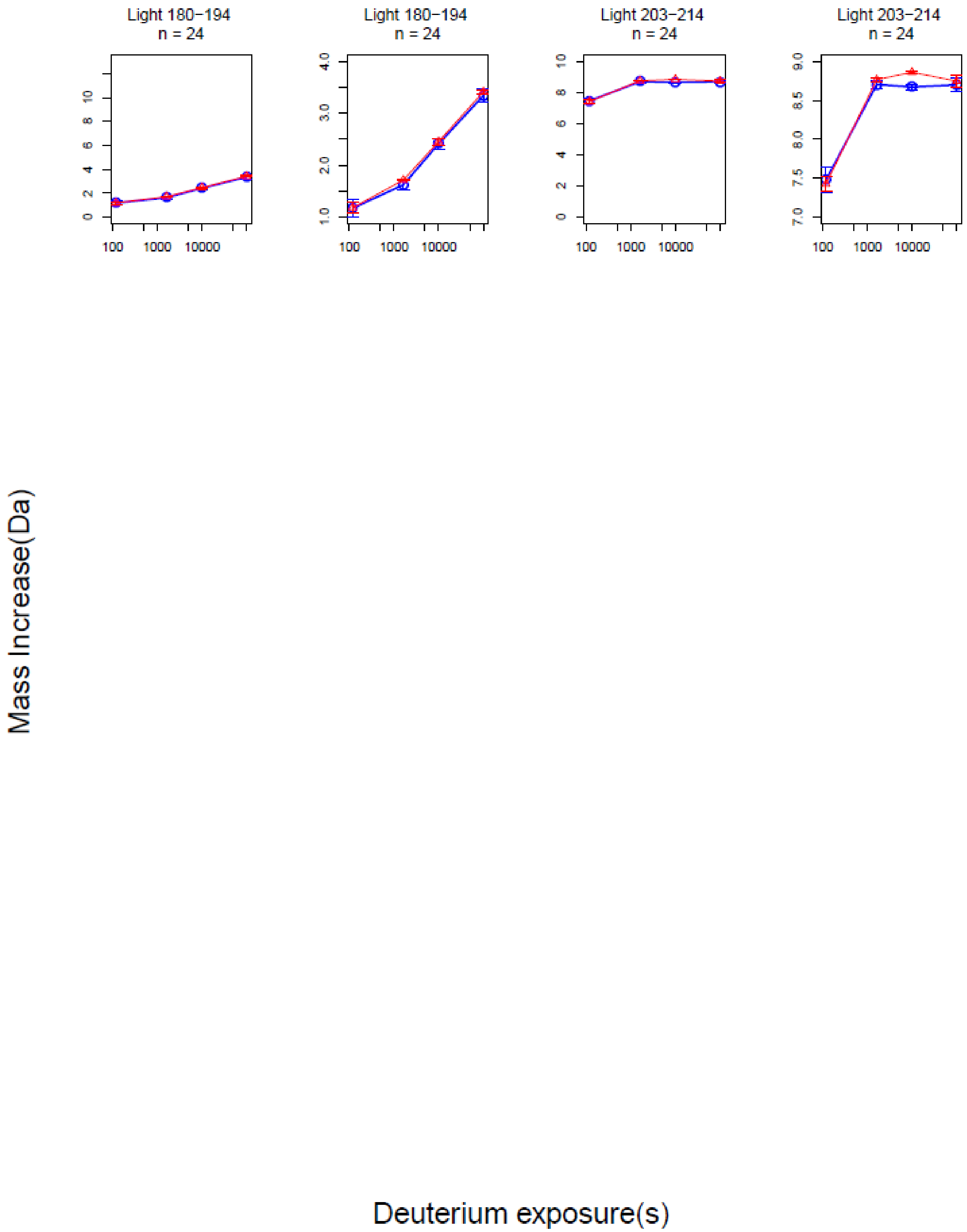
Light 180−194
n = 24
Light 180−194
n = 24
Light 203−214
n = 24
Light 203−214
n = 24
Mass Increase(Da)
Deuterium exposure(s)

Supplemental Figure S3

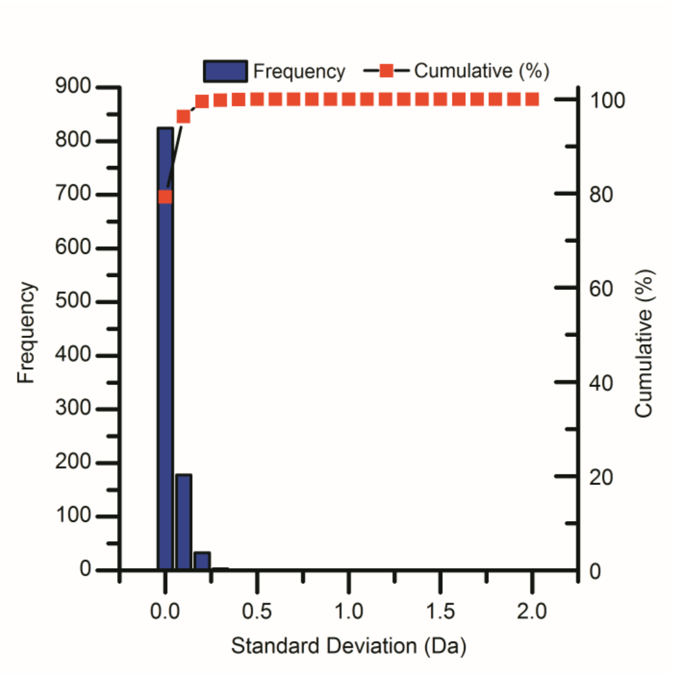

Supplemental Figure S4

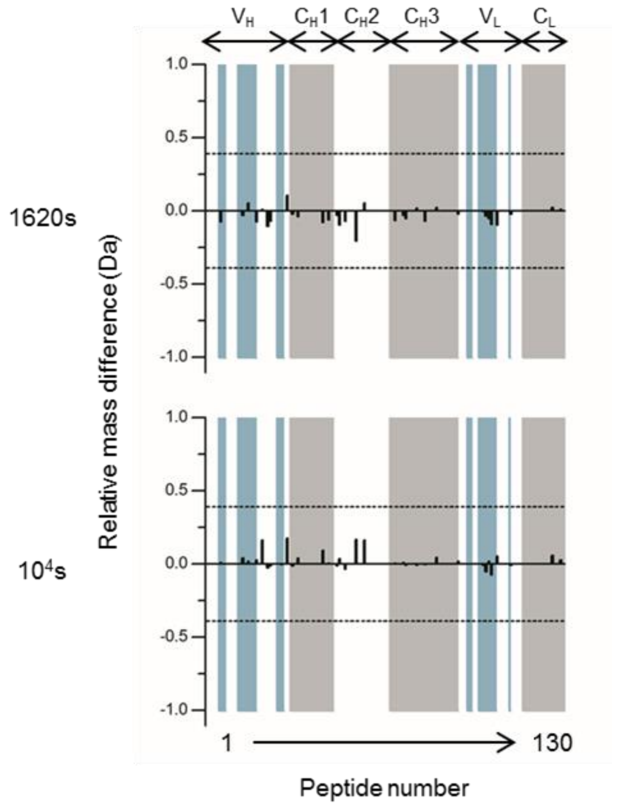

Supplemental Figure S5

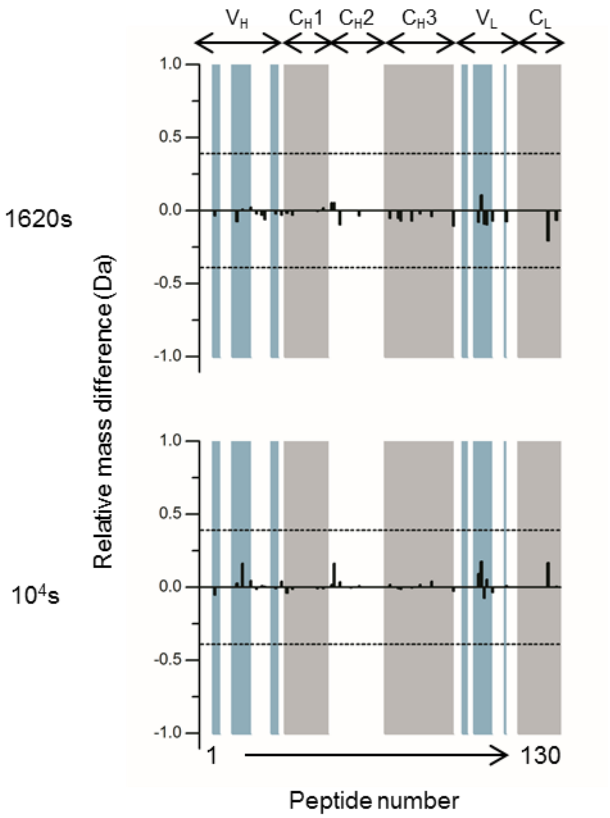

Supplement: Supplemental_Material.pdf [file kmab-07-03-1029217-s001.pdf]
